# *Pseudomonas aeruginosa* heteroresistance to levofloxacin caused by upregulated expression of essential genes for DNA replication and repair

**DOI:** 10.3389/fmicb.2022.1105921

**Published:** 2022-12-23

**Authors:** Wen-Ru Li, Zhi-Qing Zhang, Kang Liao, Bei-Bei Wang, Hui-Zhong Liu, Qing-Shan Shi, Xu-Bin Huang, Xiao-Bao Xie

**Affiliations:** ^1^Key Laboratory of Agricultural Microbiomics and Precision Application (MARA), Guangdong Provincial Key Laboratory of Microbial Culture Collection and Application, Key Laboratory of Agricultural Microbiome (MARA), State Key Laboratory of Applied Microbiology Southern China, Institute of Microbiology, Guangdong Academy of Sciences, Guangzhou, Guangdong, China; ^2^Department of Clinical Laboratory, The First Affiliated Hospital of Sun Yat-sen University, Guangzhou, Guangdong, China; ^3^Department of Pulmonary and Critical Care Medicine, The First Affiliated Hospital of Sun Yat-sen University, Guangzhou, Guangdong, China

**Keywords:** heteroresistance, levofloxacin, gene expression, fitness cost, *Pseudomonas aeruginosa*

## Abstract

*Pseudomonas aeruginosa* (*P. aeruginosa*), a common cause of severe chronic infections, has developed heteroresistance to several antibiotics, thus hindering successful treatment. In this study, we aimed to investigate the characteristics and mechanisms underlying levofloxacin (LVX) heteroresistance in *P. aeruginosa* PAS71 and PAS81 clinical isolates using a combination of physiological and biochemical methods, bacterial genomics, transcriptomics, and qRT-PCR. The six *P. aeruginosa* strains, namely PAS71, PAS72, PAS81, PAS82, ATCC27853, and PAO1, were studied. The Kirby–Bauer (K–B), minimum inhibitory concentration (MIC) test, and population analysis profile (PAP) experimental results showed that PAS71, PAS81, ATCC27853, and PAO1 were heteroresistant to LVX, with MIC of 0.25, 1, 0.5, and 2 μg/ml, respectively; PAS72 and PAS82 were susceptible to LVX with a MIC of 0.25 and 0.5 μg/ml, respectively. The resistance of PAS71 and PAS81 heteroresistant subpopulations was unstable and had a growth fitness cost. Genomic and transcriptomic results proved that the unstable heteroresistance of PAS71 and PAS81 was caused by elevated expression of essential genes involved in DNA replication and repair, and homologous recombination, rather than their genomic single-nucleotide polymorphism (SNP) and insertion–deletion (InDel) mutations. Additionally, PAS71 and PAS81 enhanced virulence and physiological metabolism, including bacterial secretion systems and biosynthesis of siderophore group nonribosomal peptides, in response to LVX stress. Our results suggest that the upregulation of key genes involved in DNA replication and repair, and homologous recombination causes unstable heteroresistance in *P. aeruginosa* against LVX. This finding provides novel insights into the occurrence and molecular regulation pathway of *P. aeruginosa* heteroresistant strains.

## Introduction

*Pseudomonas aeruginosa* (*P. aeruginosa*) is a gram-negative opportunistic pathogen with a large genome, approximately 6.3 Mb (Francis et al., 2017). Its genomic characteristics induce resistance to various antibiotics, causing severe chronic infections and hindering clinical treatments (Cao et al., 2020; Lam et al., 2020). Notably, *P. aeruginosa* has developed heteroresistance to several antibiotics, including carbapenems (Ikonomidis et al., 2008; He et al., 2018), polymyxins (Roberts et al., 2015; Lin et al., 2019), and cephalosporins (Jia et al., 2020).

Heteroresistance refers to the higher resistance level in small subpopulations than in the main bacterial population (EI-Halfawy and Valvano, 2015; Band and Weiss, 2019). Contrary to antibiotic resistance, heteroresistance is not easily detected in clinical susceptibility testing. Therefore, it affects the selection of appropriate antibiotics for successful treatment, leading to failure in clinical application of antibiotics and subsequently posing great potential threats to human health. Physiological or genetic heterogeneity could induce monoclonal heteroresistance, which is occurred and developed from a pure clone (Andersson et al., 2019). Genomic mutations usually result in stable heteroresistance, in which the resistance phenotype does not rapidly revert to susceptibility in the absence of antibiotic stress. However, genomic mutations could also induce unstable heteroresistance, a very common type of heteroresistance, in case of the mutation is intrinsically unstable and costly gene tandem amplifications or the resistance mutations confer a high fitness cost, which drive the resistant subpopulations to revert to susceptibility. In addition, overexpression of genes involved in efflux proteins could induce unstable heteroresistance (Andersson et al., 2019). Recently, a study in phenotypic and molecular characterization of heteroresistant bacteria suggested that the nature of heteroresistance is stochastical (Baaity et al., 2022).

Quinolones are a class of synthetic antibiotics widely used in clinical setting; nalidixic acid is the first member of quinolones (Hooper and Jacoby, 2015). Fluoroquinolones are developed through a key modification of the fluorine substituent at position 8 in quinolones. Levofloxacin (LVX), temafloxacin, trovafloxacin, gatifloxacin, and moxifloxacin are third-generation fluoroquinolones with an antibacterial spectrum that includes gram-positive bacteria, anaerobes, and mycobacteria (Hooper and Rubinstein, 2004).

Although there are several studies on the resistance mechanisms of bacteria against fluoroquinolones (Du et al., 2018), the heteroresistance of *P. aeruginosa* to fluoroquinolones is not well characterized. LVX is commonly used for the treatment of *P. aeruginosa* infections. In this study, we aimed to investigate the characteristics of and mechanisms underlying LVX heteroresistance in two typical heteroresistant clinical isolates of *P. aeruginosa*, PAS71 and PAS81. The results provide novel insights into the clinical treatment of *P. aeruginosa* infections.

## Materials and methods

### Strains, media, and reagents

Six *Pseudomonas aeruginosa* strains, PAS71, PAS72, PAS81, and PAS82, were isolated and identified by the Department of Clinical Laboratory, the First Affiliated Hospital of Sun Yat-sen University, and conserved in our laboratory. *P. aeruginosa* ATCC27853 was purchased from ATCC (Manassas, VA, USA) and conserved in our laboratory. *P. aeruginosa* PAO1 was provided by the CAS Key Laboratory of Pathogenic Microbiology and Immunology, Beijing, China and conserved in our laboratory. Mueller–Hinton (MH) medium and Luria-Bertani (LB) media were purchased from Guangdong Huankai Microbial Sci. and Tech. Co., Ltd. (Guangzhou, China), following our previous study (Li et al., 2021). MH agar (MHA) medium was prepared by the addition of 1.5% agar to the MH medium. Cation-adjusted Mueller–Hinton broth (CAMHB) was purchased from Beijing Solarbio Sci. Tech. Co., Ltd. (Beijing, China), which contained the following (g/L): beef dip powder, 3.0; acid hydrolyzed casein, 17.5; soluble starch, 1.5; calcium ion, 20–25 mg; magnesium ion, 10–12.5 mg; pH, 7.3 ± 0.1. MH, LB, and CAMHB media were used for aerobic cultivation at 37°C and shaken at 180 rpm. LVX susceptibility discs (Product no. 21055, 5 μg/tablet) were purchased from Beijing Bolyou Biotech. Co. Ltd. (Beijing, China). LVX (CAS: 100986–85-4, S17134-25 g, purity ≥98%) was purchased from Shanghai Yuanye Biotech. Co. Ltd. (Shanghai, China).

### Minimum inhibitory concentration determination

The minimum inhibitory concentration (MIC) of LVX against the six *P. aeruginosa* strains, PAS71, PAS72, PAS81, PAS82, ATCC27853, and PAO1, was determined using CAMHB medium according to the microdilution method (CLSI, 2020). The concentration gradients of LVX were 0.031, 0.063, 0.125, 0.25, 0.5, 1, 2, 4, 8, and 16 μg/ml. The bacterial concentration was 5 × 10^5^ colony-forming units (CFUs)/mL. The LVX concentration was 0 μg/ml, while bacterial concentration was 0 CFU/ml in the control (+) and control (−) groups. The cultures were incubated at 37°C for 18 h in a Multiskan Sky full-wavelength microplate reader (Thermo Scientific, Waltham, MA USA), and the OD_600_ was determined. According to CLSI (2020), the MIC breakpoints for *P. aeruginosa* to LVX are as follows: susceptible strain (≤1 μg/ml), intermediate strain (2 μg/ml), and resistant strain (≥4 μg/ml).

### Kirby–Bauer test

The Kirby–Bauer (K–B) test for the six strains was performed according to the Clinical and Laboratory Standards Institute (CLSI) (2020). Molten sterilized MHA medium (10 ml) was poured into 12 sterilized Petri dishes. A bacterial cell suspension (50 μl) in the log phase containing approximately 5 × 10^6^ CFU was streaked over the MHA surface using a sterilized cotton swab for uniform cell growth. Three LVX susceptibility discs (5 μg/tablet) were attached to the surface of the MHA medium. The Petri dishes were incubated at 37°C for 18 h, and the diameter of the LVX disc inhibition circles and heteroresistant activity were determined. According to CLSI (2020), the zone diameter breakpoints of *P. aeruginosa* to 5 μg LVX discs are as follows: susceptible strain (≥22 mm), intermediate strain (15–21 mm), and resistant strain (≤14 mm).

### Population analysis profile test

The population analysis profile (PAP) test for the six strains was modified according to a previous study (Nicoloff et al., 2019). MHA plates containing 0 (Blank) and 0.016–16 μg/ml gradient-diluted LVX were prepared. A bacterial suspension containing 10^8^–10^2^ CFU/ml cells was prepared. The bacterial suspension of 100 μl was coated on the LVX gradient-diluted plates in triplicate. The plates were incubated at 37°C for 48 h, and the colonies were then counted. PAP curves were drawn based on the colony count and corresponding LVX concentrations.

### Fitness cost determination

Four resistant subpopulation isolates from the PAS71 strain were selected from the 1 μg/ml aforementioned plates and labeled as PAS71-1, PAS71-2, PAS71-3, and PAS71-4. Four resistant subpopulation isolates from the PAS81 strain were selected from 4 μg/ml aforementioned plates and labelled as PAS81-1, PAS81-2, PAS81-3, and PAS81-4. Glycerol tubes were prepared to preserve resistant subpopulation isolates at-20°C. The bacterial suspensions of PAS71, PAS81, and the eight resistant subpopulation isolates were prepared in the log phase. MH medium (200 μl) and the bacterial suspension were added to a 96-well plate at a concentration of 10^6^ CFU/ml. The plate was incubated at 37°C for 48 h in the Multiskan Sky full-wavelength microplate reader, and OD_600_ was determined every hour. The growth curves of the PAS71 and PAS81 parental strains and the eight resistant subpopulation isolates were plotted based on OD_600_.

### Resistance stability determination

The aforementioned strains and subpopulation isolate bacterial suspensions were prepared in log phase. The eight resistant subpopulation isolates were sub-cultured in an antibiotic-free MH medium according to a 1% inoculation amount. They were then incubated in the Multiskan Sky full-wavelength microplate reader at 37°C and 180 rpm for 18 h and sub-cultured continuously for 50 generations. The MIC of the PAS71 and PAS81 parent strains and their four resistant subpopulation isolates were determined using a CAMHB medium.

### Genomic analysis

The DNA of five strains of *P. aeruginosa*, namely PAS71, PAS81, PAS82, ATCC27853, and PAO1, was extracted using the DNeasy UltraClean Microbial Kit (Qiagen, USA), following the manufacturer′s instructions. The DNA concentration was detected using a Qubit fluorometer (Thermo Fisher Scientific, MA, USA). DNA integrity and purification were assessed using agarose gel electrophoresis. DNA (> 1 ng) was randomly fragmented using Covaris LE220. The fragmented DNA was tested using an Agilent 2,100 and purified using the Agencourt AMPure XP kit (Beckman Coulter, Miami, FL, USA). The selected fragments were subjected to end-repair, 3′ adenylation, adapter ligation, and purified using the Agencourt AMPure XP kit. The double-stranded PCR products were heat-denatured and circularized using the splint oligo sequence. Single-stranded circular DNA was formatted as the final library and qualified *via* quality control (QC). Qualified libraries were sequenced on the BGISEQ-500 platform (BGI-Shenzhen, China).

Statistical analysis of the data and downstream bioinformatics analysis were performed on filtered, high-quality data, referred to as clean data. The reference genome was *P. aeruginosa* PAO1 from the National Center for Biotechnology Information (NCBI) database.^1^ Single-nucleotide polymorphism (SNP) and insertion–deletion (InDel) were identified using the HaplotypeCaller of Genome Analysis Toolkit (GATK) and annotated using SnpEff software. The dataset was provided by the NCBI Sequence Read Archive database (PRJNA781681 and PRJNA891088).

### Transcriptomic analysis

LB medium (100 ml) was inoculated with the exponential growth phase PAS71 and PAS81 strains at 10^8^ CFU/ml. LVX solution (0.125 mg/ml) was then added at a final concentration of 0 (control) or 0.125 μg/ml, in triplicate. All 12 experimental groups were incubated in a water bath shaker at 37°C and 180 rpm for 5 h. The cells were then sampled and centrifuged at 4°C, 4,000 rpm for 5 min. The cell precipitates in the control and LVX-treated groups were snap-frozen at −80°C.

As previously described, RNA sample extraction, library construction, high-throughput sequencing, and enrichment analysis were performed (Li et al., 2021). Transcriptomic sequencing was performed using the BGI Genomics software (Shenzhen, China). To evaluate the phenotype variation, the Kyoto Encyclopedia of Genes and Genomes (KEGG) and Gene Ontology (GO) enrichment analyses of significant differentially expressed genes (DEGs) were performed based on a hypergeometric test. Significance levels were corrected with a rigorous threshold of *q* ≤ 0.05. The transcriptomic dataset was provided in the NCBI, Gene Expression Omnibus database (GSE217409 and GSE189021).

### Quantitative real-time polymerase chain reaction validation

In the transcriptomic analysis, 2 LVX target genes of *gyrB* (PA0004) and *gyrA* (PA3168) in both PAS71 and PAS81 strains and 14 significant DEGs were selected for quantitative real-time polymerase chain reaction (qRT-PCR) verification, including 7 DEGs in the PAS71 strain [*recA* (PA3617), *uvrD* (PA5443), *xseB* (PA4042), *ssb* (PA4232), *mutM* (PA0357), *crc* (PA5332), and *rhlA* (PA3479)] and 7 DEGs in the PAS81 strain [*recA* (PA3617), *gspD* (PA0685), *vgrG1* (PA0091), *hcpC* (PA0263), *clpV1* (PA0090), *ppkA* (PA0074), and PA4889]. The PAS71 and PAS81 culture conditions, control, and LVX treatment experiments followed the above methods. The LVX concentrations were 0 (control) and 0.125 μg/ml. Total RNA extraction, PCR primer design and synthesis, and experimental procedure were the same as previously described (Li et al., 2021). The 16S rRNA gene was used as an endogenous reference. The primer sequences are listed in Supplementary Table S3 of Supplementary Material 1.

### Biofilm validation

For biofilm validation, the PAS71 and PAS81 culture conditions and the control and LVX treatment experiments were set up as previously described (Li et al., 2021). All experimental groups were incubated at 37°C for 24 h. The biofilm determination was in accordance with a previously described method (Li et al., 2021), and two dependent experiments were carried out in triplicate.

### Statistical analysis

Two independent experiments were performed in triplicate for each assay. The error bars in the figures indicate standard deviation (SD), and data represent mean ± SD. Statistical significance was determined using the independent Student′s *t*-test, with value of *p* < 0.01 denoting an extremely significant difference and value of *p* < 0.05 denoting a significant difference.

## Results

### MIC results

The MIC breakpoints of *P. aeruginosa* to LVX are listed in Table 1 according to the CLSI (2020); Table 1 also shows that MICs of LVX against the six strains, namely ATCC27853, PAO1, PAS71, PAS72, PAS81, and PAS82 were 0.5, 2, 0.25, 0.25, 1, and 0.5 μg/ml, respectively. Thus, the five *P. aeruginosa* strains ATCC27853, PAS71, PAS72, PAS81, and PAS82 were susceptible to LVX, while the PAO1 strain was intermediate to LVX.

**Table 1:** Minimum inhibitory concentration (MIC) and Kirby–Bauer (K–B) test results of levofloxacin against the six strains of *Pseudomonas aeruginosa.*

Strains	Interpretive categories and MIC breakpoints, μg/mL			K–B disc content	Interpretive categories and zone diameter breakpoints, nearest whole mm		
	Susceptible	Intermediate	Resistant		Sensitive	Intermediate	Resistant
*P. aeruginosa* breakpoints	≤1	2	≥4	5 μg	≥22	15–21	≤14
*P. aeruginosa* ATCC27853	0.5			5 μg	22		
*P. aeruginosa* PAO1		2		5 μg		15	
*P. aeruginosa* PAS71	0.25			5 μg	30		
*P. aeruginosa* PAS72	0.25			5 μg	30		
*P. aeruginosa* PAS81	1			5 μg	27		
*P. aeruginosa* PAS82	0.5			5 μg	26		

### K–B results

**Figure 1:**
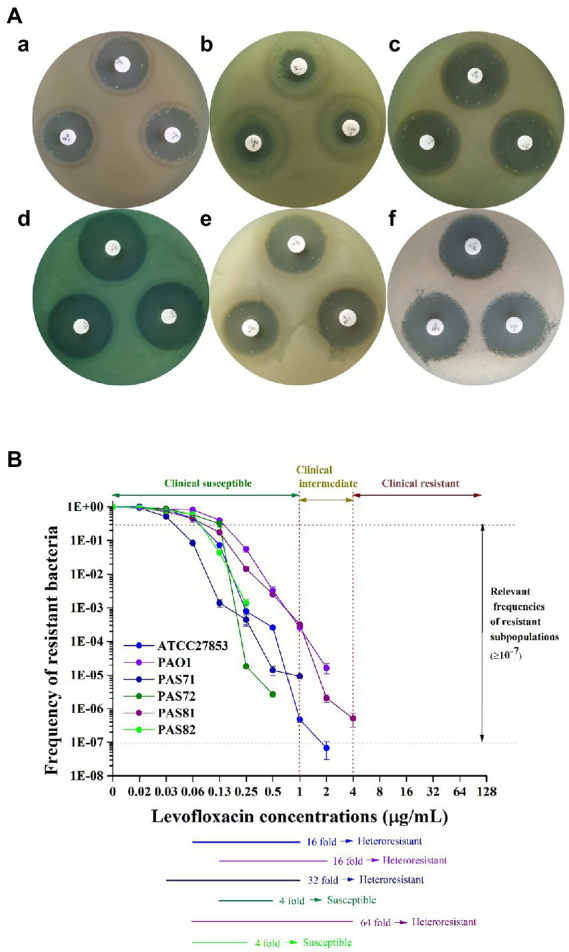
Photos and curves of heteroresistance characteristics of six *Pseudomonas aeruginosa* strains to LVX. **(A)** Kirby–Bauer (K–B) test. a, *P. aeruginosa* ATCC27853, heteroresistant to LVX; b, *P. aeruginosa* PAO1, heteroresistant to LVX; c, *P. aeruginosa* PAS71, heteroresistant to LVX; d, *P. aeruginosa* PAS72, susceptible to LVX; e, *P. aeruginosa* PAS81, heteroresistant to LVX; f, *P. aeruginosa* PAS82, susceptible to LVX. **(B)** Schematic population analysis profile (PAP) curves. The clinical susceptible, intermediate, and resistant levels of *P. aeruginosa* to LVX are indicated by two red vertical dotted lines. The area between the two horizontal gray dotted lines suggest that the resistance frequencies of the subpopulations are ≥1 × 10^−7^. Six horizontal-colored lines highlighted the PAP test results of the six *P. aeruginosa* strains from the maximum concentration that did not inhibit growth of the main subpopulations to the end of the concentration when the resistance frequency of the subpopulations was ≥1 × 10^−7^.

The zone diameter breakpoints of *P. aeruginosa* to 5 μg LVX discs are provided in Table 1 according to CLSI (2020). Photographs of the plate culture for the K–B test show a clear bacteriostatic circle around each LVX disc in the PAS72 (Figures 1A-d) and PAS82 (Figures 1A-f) groups. There was scarce colony growth within the circles, and the average diameter was 30 mm in the PAS72 group and 26 mm in the PAS82 group, indicating that both strains are susceptible to LVX. Several colonies were present in each bacteriostatic circle around the LVX discs, with an average diameter of 22 and 30 mm in the ATCC27853 (Figures 1A-a) and PAS71 groups (Figures 1A-c), respectively, indicating susceptibility to LVX, with several heteroresistant subpopulation colonies. The PAS81 group (Figures 1A-e) had dozens of colonies in each bacteriostatic circle, with an average diameter of 27 mm, indicating susceptibility to LVX but with many heteroresistant subpopulation colonies. The PAO1 group (Figures 1A-b) had a small bacteriostatic circle, with an average diameter of 15 mm, around each LVX disc, indicating that this strain is intermediate to LVX. Therefore, *P. aeruginosa* PAS72 and PAS82 are susceptible to LVX; ATCC27853, PAS71, and PAS81 are susceptible to LVX but with some heteroresistant subpopulation colonies; and *P. aeruginosa* PAO1 is intermediate to LVX.

### PAP results

The PAP test curves for the six *P. aeruginosa* strains (ATCC27853, PAO1, PAS71, PAS72, PAS81, and PAS82) exposed to LVX are shown in Figure 1B. Heteroresistant strains have been defined as heteroresistant subpopulations capable of growth at antibiotic concentrations approximately 8-fold higher than the highest concentration without affecting the dominant population’s growth; they have frequencies greater than 1 × 10^−7^, according to previous reports (EI-Halfawy and Valvano, 2015; Andersson et al., 2019; Nicoloff et al., 2019). Figure 1B shows that the resistant bacterial frequency of all six *P. aeruginosa* strains gradually decreased with increase in antibiotic concentration from 0.016 to 4 μg/ml, indicating that they were not resistant to LVX. The highest LVX concentrations that had no effect on the growth of the dominant population (the highest non inhibitory concentration, HNIC) of ATCC27853, PAO1, PAS71, PAS72, PAS81, and PAS82 strains were 0.063, 0.125, 0.031, 0.125, 0.063, and 0.063 μg/ml, respectively. The frequency of resistant cells of PAS72 and PAS82 strains was higher than 1 × 10^−7^ at LVX concentrations of 0.5 μg/ml and 0.25 μg/ml, respectively, which were 4-fold higher than their HNIC. The frequencies of resistant cells of the ATCC27853 and PAO1 strains were higher than 1 × 10^−7^ at LVX concentrations of 1 μg/ml and 2 μg/ml, respectively, which were 16-fold higher than their HNIC. The frequencies of resistant cells of the PAS71 and PAS81 strains were higher than 1 × 10^−7^ at LVX concentrations of 1 and 4 μg/ml, which were 32-and 64-fold higher than their HNIC, respectively. The concentrations of resistant subpopulations of all four *P. aeruginosa* strains, except PAS72 and PAS82, at frequencies higher than 1 × 10^−7^ were eight times higher than their HNIC. According to the previous definition of heteroresistance, with the exception of PAS72 and PAS82 strains that were susceptible to LVX, the other four strains (ATCC27853, PAO1, PAS71, and PAS81) were considered heteroresistant to LVX. However, the concentration of only the PAS81 strain (4 μg/ml) resistant subpopulation at a frequency ≥1 × 10^−7^ reached the level for clinical resistance (≥4 μg/ml). In contrast, the concentrations of the resistant subpopulation from the other three strains, ATCC27853 (1 μg/ml), PAO1 (2 μg/ml), and PAS71 (1 μg/ml) at frequencies ≥1 × 10^−7^ were all lower than the level of clinical resistance (≥ 4 μg/ml).

### Fitness cost

**Figure 2:**
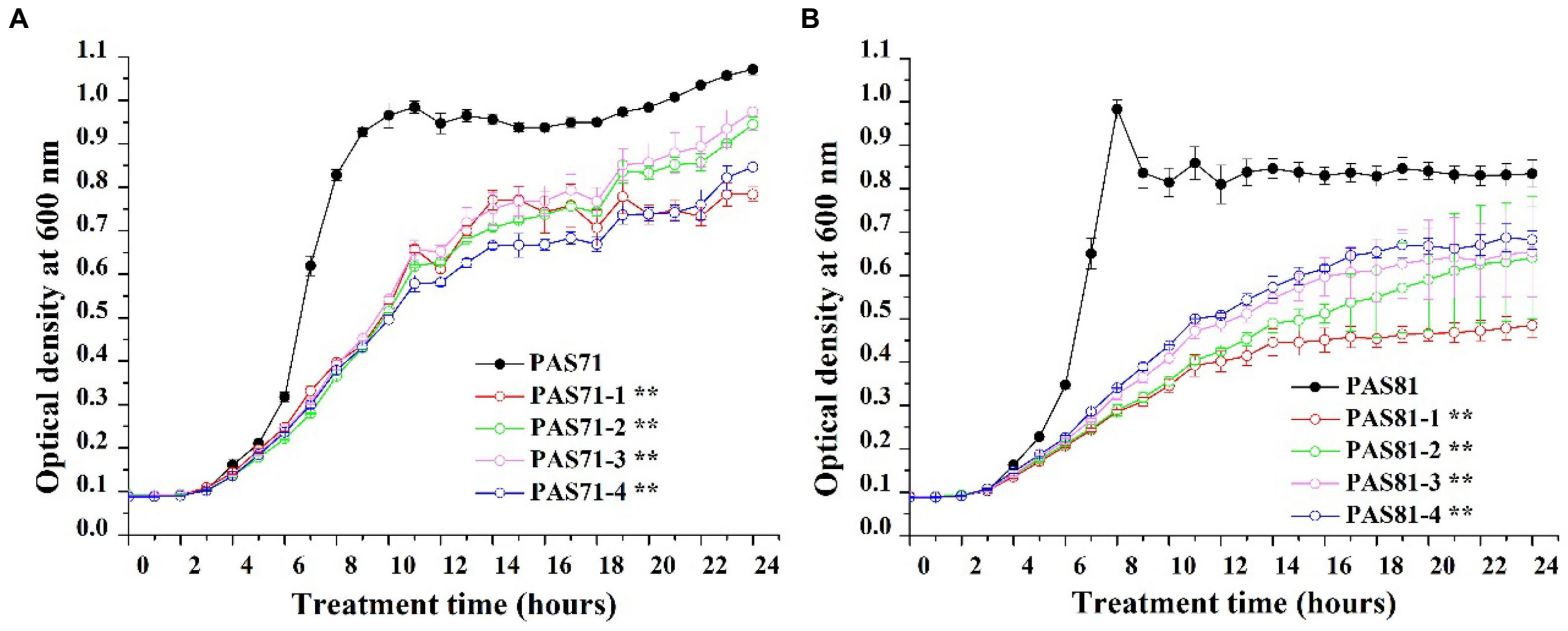
Growth curves of *Pseudomonas aeruginosa* PAS71 and PAS81 parental strains and their four heteroresistant subpopulation isolates. **(A)** The *P. aeruginosa* PAS71 parental strain and its four heteroresistant subpopulation isolates. **(B)** The *P. aeruginosa* PAS81 parental strain and its four heteroresistant subpopulation isolates. *k* value denotes slope of growth curve cultured for 6 to 10 h; ** indicates an extremely significant difference in *k* value with *p* < 0.05 compared with the parental train based on the independent Student’s *t*-test.

Growth curves of the PAS71 and PAS81 parental strains and their four resistant subpopulation isolates are shown in Figure 2. All growth curves of the PAS71 parental strain and its four resistant subpopulation isolates showed a typical bacterial growth curve, including the lag, exponential, and stationary phases (Figure 2A). The *k* values (slope of growth curves) and their independent Student′s *t*-tests were analyzed between the PAS71 parental strain and their four heteroresistant subpopulation isolates based on data from their exponential growth phase of incubation for 4–8 h. The exponential growth phase rates of all four heteroresistant subpopulation isolates of PAS71-1 (*k* = 0.05), PAS71-2 (*k* = 0.05), PAS71-3 (*k* = 0.05), and PAS71-4 (*k* = 0.05) were significantly lower than that of the PAS71 parental strain (*k* = 0.13), with *p* < 0.01. This indicates that all four subpopulation isolates grew at a much slower rate than the PAS71 parental strain.

Similarly, all growth curves of PAS81 and its four resistant subpopulation isolates showed a typical bacterial growth curve, including the lag, exponential, and stationary phases (Figure 2B). The *k* values and their independent Student′s *t*-tests were analyzed between the PAS81 parental strain and their four heteroresistant subpopulation isolates based on data from their exponential growth phase of incubation for 4–8 h. The exponential growth phase rates of all four heteroresistant subpopulation isolates of PAS81-1 (*k* = 0.03), PAS81-2 (*k* = 0.03), PAS81-3 (*k* = 0.04), and PAS81-4 (*k* = 0.04) were significantly lower than that of the PAS81 parental strain (*k* = 0.16), with *p* < 0.01. This indicates that all four subpopulation isolates grew at a much slower rate than the PAS81 parental strain.

Therefore, the growth of all heteroresistant subpopulation isolates of PAS71 and PAS81 incurs a growth fitness cost.

### Resistant stability

**Figure 3:**
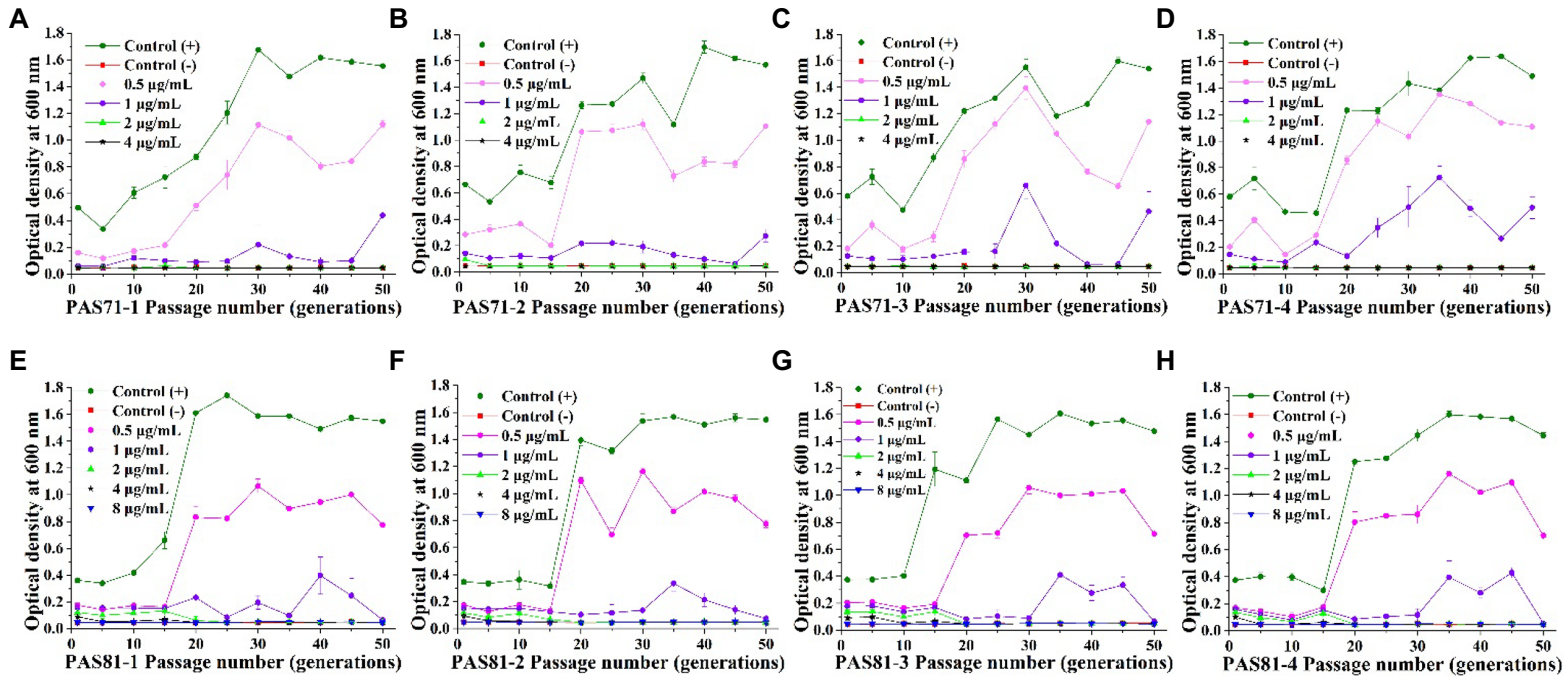
Curves of levofloxacin resistance of the four subpopulation isolates of PAS71 and PAS81 after 50 generations of subcultures without antibiotics. **(A)** PAS71-1; **(B)** PAS71-2; **(C)** PAS71-3; **(D)** PAS71-4; **(E)** PAS81-1; **(F)** PAS81-2; **(G)** PAS81-3; **(H)** PAS81-4.

The eight PAS71 heteroresistant subpopulation isolates (PAS71-1, PAS71-2, PAS71-3, and PAS71-4) and PAS81 heteroresistant subpopulation isolates (PAS81-1, PAS81-2, PAS81-3, and PAS81-4) were sub-cultured continuously for 50 generations without antibiotic stress. The MICs of LVX against the four PAS71 and PAS81 heteroresistant subpopulation isolates were determined for the 1^st^ to 50^th^ generations; MIC curves based on the determined optical density at 600 nm are shown in Figures 3A–H.

The MICs of the first generation of PAS71-1, PAS71-2, PAS71-3, and PAS71-4 resistant subpopulation isolates were 2, 4, 2, and 2 μg/ml, respectively (Figures 3A–D). Moreover, the MIC of PAS71-2 quickly reverted to 2 μg/ml after passage for five generations without antibiotics. After passage without antibiotics for 50 generations, the MIC of all four PAS71 resistant subpopulation isolates remained at 2 μg/ml.

The MICs of the first generation of all four PAS81 resistant subpopulation isolates were 8 μg/ml (Figures 3E–H). However, the MIC of all four PAS81 resistant subpopulation isolates gradually reverted to 2 μg/ml after continuous passage without antibiotics. The MIC of PAS81-3 reverted to 4 μg/ml in the 10th generation and 2 μg/ml in the 20th generation. The MIC of the remaining resistant subpopulation isolates (PAS81-1, PAS81-2, and PAS81-4) reverted to 4 μg/ml in the 5th generation and 2 μg/ml in the 20th generation. Therefore, resistance stability of the four *P. aeruginosa* PAS81 resistance subpopulation isolates was unstable, and their MICs reverted to 2 μg/ml from the initial 8 μg/ml until passage without antibiotics for 50 generations.

The growth rates of *P. aeruginosa* PAS71 and PAS81 resistant subpopulation isolates showed an increasing trend after continuous passage without antibiotics (Figure 3). The growth rates (0.3–0.7 of OD_600_, unexposed to antibiotics) of all initial resistant subpopulation isolates were much lower than their PAS71 and PAS81 parental strains (1.3–1.7 of OD_600_, unexposed to antibiotics). After continuous passage without antibiotics for more than 25 generations, the growth rates of the resistant subpopulation isolates reverted to their parental strains levels. Therefore, all resistant subpopulation isolates of PAS71 and PAS81 showed growth fitness costs; their growth rates reverted to their parental strains level after continuous passage without antibiotics for more than 25 generations. Moreover, their resistance was unstable as their MICs reverted to 2 μg/ml from the initial 4 μg/ml (PAS71-2) or 8 μg/ml (all four PAS81 resistance subpopulation isolates) after continuous passage without antibiotics for 50 generations.

### Genomic results

#### SNP mutation

Supplementary Table S1 of Supplementary Material 1 shows the SNP analysis results based on the comparison between genome data of the five sequenced strains with the reference genome of *P. aeruginosa* PAO1 from the NCBI database. The nonsynonymous SNPs of *P. aeruginosa* PAS71, PAS81, PAS82, ATCC27853, and PAO1 were 5,518, 8,540, 6,191, 5,231, and 909, respectively. The numbers of nonsynonymous SNPs in the PAO1 strain were the lowest among the four experimental strains as *P. aeruginosa* PAO1 was the same strain as the reference. The detailed SNP mutation information of all five strains is shown in Supplementary Material 2. The genes of *gyrB* (PA0004) and *gyrA* (PA3168) are the target genes of LVX. The genomic results showed some SNPs in *gyrB* of PAS81 (18 SNPs) and PAS82 (nine SNPs) strains, but none in strains PAS71, ATCC27853, and PAO1 or in *gyrA* of all five strains. The gyrB of both PAS81 and PAS82 had only one and same nonsynonymous SNP, while the other SNPs were all synonymous. The detailed information of SNPs in *gyrB* of the PAS81 strain is provided in Table 2. The nonsynonymous SNP mutation in *gyrB* of the PAS81 and PAS82 strains is “A to G” at sequence position 4,650, which resulted in codon mutation of “AAC to GAC” and amino acid mutation of asparagine (N) to aspartic acid (D). The synonymous SNPs in *gyrB* of the PAS81 and PAS82 strains have no biological significance. The susceptible PAS82 and heteroresistant PAS81 strains had one similar nonsynonymous mutation, indicating that the nonsynonymous SNP mutation in *gyrB* was not associated with the heteroresistance of PAS81 strain.

**Table 2:** Single-nucleotide polymorphism (SNP) and insertion–deletion (InDel) gene information of *gyrB* (PA0004) and *gyrA* (PA3168) of *Pseudomonas aeruginosa* PAS81 based on bacterial genome sequencing.

Ref_id	Site	Ref_base ↔ sample base	Snp_ status	Strand	Codon_ mutate	aa_ mutate	Mutate _type	Gene _id	Pos_ start	Pos_ end
AE004091.2	4445	C ↔ T	Homo	+	TAC ↔ TAT	Y ↔ Y	Syn	PA0004	4275	6695
AE004091.2	4650	A ↔ G	Homo	+	AAC ↔ GAC	N ↔ D	Nonsyn	PA0004	4275	6695
AE004091.2	4670	A ↔ G	Homo	+	CTA ↔ CTG	L ↔ L	Syn	PA0004	4275	6695
AE004091.2	4772	C ↔ T	Homo	+	GGC ↔ GGT	G ↔ G	Syn	PA0004	4275	6695
AE004091.2	4856	G ↔ A	Homo	+	GAG<- > GAA	E ↔ E	Syn	PA0004	4275	6695
AE004091.2	5045	C ↔ T	Homo	+	GCC ↔ GCT	A ↔ A	Syn	PA0004	4275	6695
AE004091.2	5111	C ↔ T	Homo	+	GAC ↔ GAT	D ↔ D	Syn	PA0004	4275	6695
AE004091.2	5126	G ↔ C	Homo	+	CTG ↔ CTC	L ↔ L	Syn	PA0004	4275	6695
AE004091.2	5186	G ↔ A	Homo	+	CTG ↔ CTA	L ↔ L	Syn	PA0004	4275	6695
AE004091.2	5195	G ↔ A	Homo	+	AAG ↔ AAA	K ↔ K	Syn	PA0004	4275	6695
AE004091.2	5321	T ↔ C	Homo	+	ACT<- > ACC	T ↔ T	Syn	PA0004	4275	6695
AE004091.2	5588	C ↔ T	Homo	+	CGC ↔ CGT	R ↔ R	Syn	PA0004	4275	6695
AE004091.2	5726	A ↔ G	Homo	+	GAA ↔ GAG	E ↔ E	Syn	PA0004	4275	6695
AE004091.2	5975	C ↔ T	Homo	+	AGC ↔ AGT	S ↔ S	Syn	PA0004	4275	6695
AE004091.2	6014	G ↔ A	Homo	+	GCG ↔ GCA	A ↔ A	Syn	PA0004	4275	6695
AE004091.2	6209	C ↔ T	Homo	+	TCC ↔ TCT	S ↔ S	Syn	PA0004	4275	6695
AE004091.2	6504	C ↔ T	Homo	+	CTG ↔ TTG	L ↔ L	Syn	PA0004	4275	6695
AE004091.2	6587	T ↔ C	Homo	+	GAT ↔ GAC	D ↔ D	Syn	PA0004	4275	6695
**Ref_id**	**Site**	**Base**	**Ho/He**	**Strand**	**Pos_type**	**Type**	**Annot _type**	**Gene _id**	**Pos_ start**	**Pos_ end**
AE004091.2	3556459	CTCGGA	Homo	−	Gene_inner	D6	CDS	PA3168	3556427	3559198

*Ref_id, reference id; Ref_base* ↔ *sample base, reference base* ↔ *sample base; Snp_status, SNP status; Codon_mutate, condon mutation; aa-mutate, amino acid mutate; Mutate_type, mutation type; Gene_id, gene id; Pos_start, start position; Pos_end, end position; Syn, synonymous; Nonsyn, nonsynonymous; Ho/He, homozygote/heterozygote; Annot_type, annotation type; CDS, coding sequences*.

#### InDel mutation

The InDel analysis results obtained by comparing the genome data of the five sequenced strains with the reference are shown in Supplementary Table S2 of Supplementary Material 1. The total number of InDels in the coding sequence of *P. aeruginosa* strains, PAS71, PAS81, PAS82, ATCC27853, and PAO1, were 74, 121, 76, 130, and 20, respectively. The genomic results showed one InDel mutation in *gyrA* (PA3168) of the PAS81 strain, but none in PAS71, PAS82, ATCC27853, and PAO1 strains or in *gyrB* of all five strains. The detailed InDel information in *gyrA* of the PAS81 strain is provided in Table 2. The *gyrA* gene of the PAS81 strain had a deletion mutation of “CTCGGA” in sequence position of 3,556,459.

The genomic analysis indicated that PAS81 had one nonsynonymous SNP in *gyrB* and one six-base deletion mutation in *gyrA*; the PAS82 strain had one similar nonsynonymous SNP mutation in *gyrB*, while the PAS71, ATCC27853, and PAO1 strains had no SNP and InDel mutations. Therefore, the heteroresistance of *P. aeruginosa* PAS71 to LVX was not related to *gyrB* and gyrA mutations and that of PAS81 was not related to *gyrA* mutation.

### Transcriptomic results

#### Volcano plots

**Figure 4:**
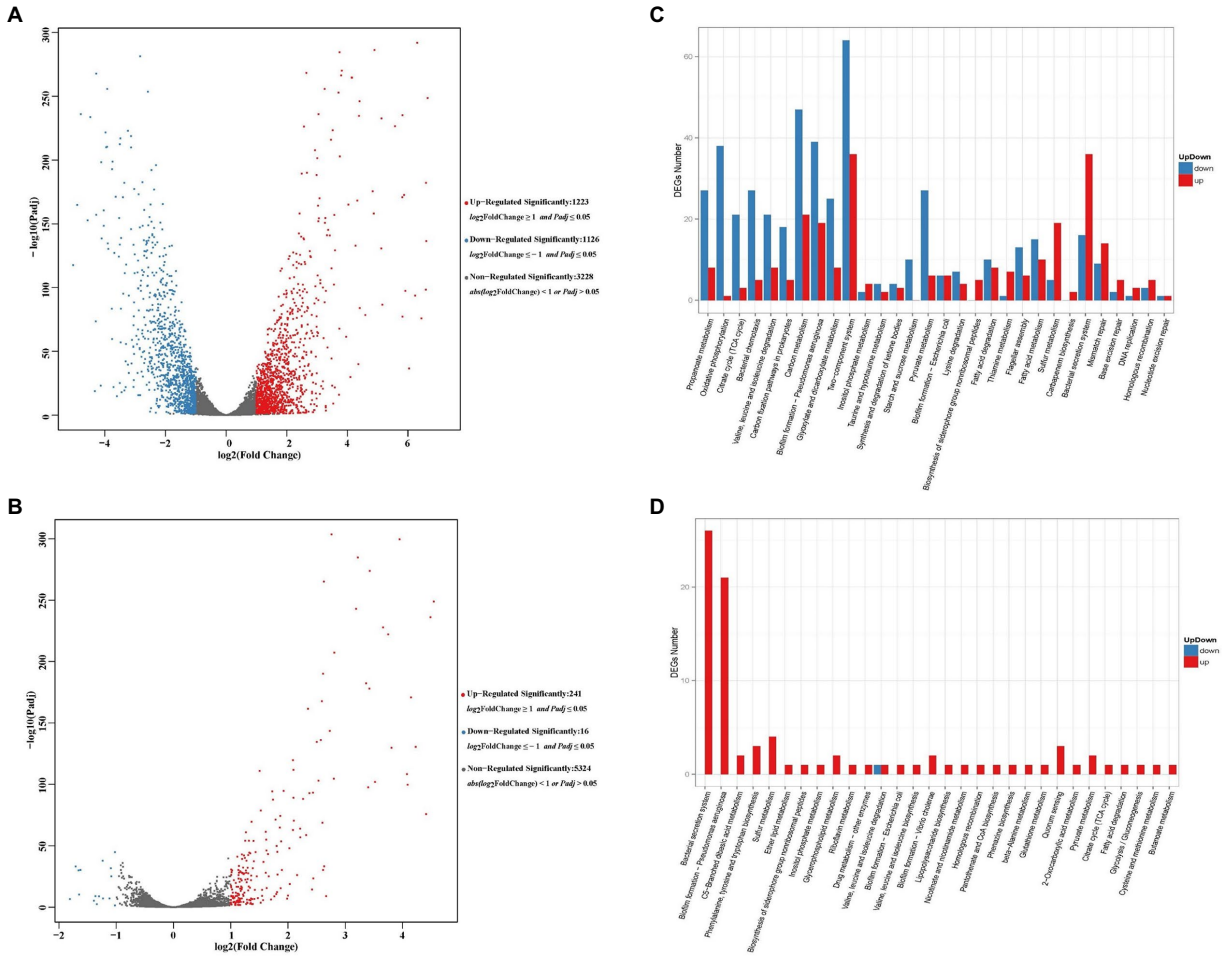
Volcano plots and KEGG maps of differentially expressed genes (DEGs) for untreated and 0.125 μg/ml levofloxacin-treated *Pseudomonas aeruginosa* PAS71 and PAS81 for 5 h based on RNA-seq. **(A,B)** Volcano plots of PAS71 and PAS81 strains, respectively. A dot in the graph represents a gene. The x-axis and y-axis represent log2 of the fold change and t-statistic as-log10 of the value of *p*, respectively. The genes depicted in red (upregulated) and green (downregulated) are differentially expressed genes with >2-fold change and a value of *p* < 0.05. **(C,D)** KEGG column maps of PAS71 and PAS81 strains, respectively. Thirty main pathways in each KEGG map show the up-and down DEG numbers (0.00 < *q* < 0.05).

A total of 5,577 genes were detected in *P. aeruginosa* PAS71 RNA-seq analysis and 5,581 in PAS81 RNA-seq analysis, of which, 2,349 genes were significantly differentially expressed, 3,228 genes were not differentially regulated in the PAS71 strain; 257 genes were significantly differentially expressed, and 5,324 genes were not differentially regulated in the PAS81 strain. Two maps of transcriptomic volcano plots for PAS71 and PAS81 strains are shown in Figures 4A,B, respectively. Figure 4A shows 1,223 upregulated genes and 1,126 downregulated genes in the PAS71 volcano plots. Figure 4B shows that the number of upregulated and downregulated genes of the PAS81 strain varied significantly. There were 241 upregulated genes and only 16 downregulated genes in the PAS81 volcano plots. The experimental results indicated that the antibacterial effect of 0.125 μg/ml LVX on the PAS71 strain was much greater than that on PAS81 strain.

#### KEGG enrichment

The DEG enrichment of PAS71 and PAS81 RNA-seq was analyzed in the KEGG database. The up-down KEGG enrichment maps are shown in Figures 4C,D for the KEGG pathway enrichment of DEGs in the PAS71 and PAS81 RNA-seq, respectively.

The PAS71 up-downregulated genes exposed to 0.125 μg/ml LVX were enriched in 30 main pathways (Figure 4C). Five pathways are related to LVX resistance, including mismatch repair, base excision repair, DNA replication, homologous recombination, and nucleotide excision repair. The remaining 25 up-downregulated pathways were related to *P. aeruginosa* virulence and physiological metabolism, such as biofilm formation, biosynthesis of siderophore group nonribosomal peptides, and bacterial secretion systems related to bacterial virulence. The eight detailed KEGG pathways of PAS71 RNA-seq, including mismatch repair (Supplementary Figure S1A), base excision repair (Supplementary Figure S1B), DNA replication (Supplementary Figure S1C), homologous recombination (Supplementary Figure S1D), nucleotide excision repair (Supplementary Figure S1E), biofilm formation (Supplementary Figure S1F), biosynthesis of siderophore group nonribosomal peptides (Supplementary Figure S1G), and the bacterial secretion system (Supplementary Figure S1H) are shown in Supplementary Figure S1 in the Supplementary Material 2.

The five KEGG pathways shown in Supplementary Figure S1A–E are related to DNA replication and repair, and homologous recombination. In these pathways, the expression level of 12 key genes encoding helicase II UvrD (*uvrD*, PA5443), single-stranded DNA-binding protein SSB (*ssb*, PA4232), DNA polymerase III subunits gamma/tau (*dnaX*, PA1532), recombinase RecA (*recA*, PA3617), DNA repair protein RecO (*recO*, PA0772), DNA primase DnaG (*dnaG*, PA0577), exonucleases ExoVII (*xseA*, PA3777; *xseB*, PA4042) and single-stranded-DNA-specific exonuclease RecJ (*recJ*, PA3725), bifunctional glycosylase Fpg (*mutM*, PA0357), monofunctional glycosylase MPG (PA4010), and AP-endonuclease Xth (*crc*, PA5332) of the PAS71 strain were all upregulated following exposure to 0.125 μl/ml LVX. However, the expression level of the five genes encoding DpoIII (PA3232), bifunctional glycosylase NTH (*nth*, PA3495), and exonucleases RecB (*recB*, PA4284), RecD (*recD*, PA4283), and UvrB (*uvrB*, PA3138) were downregulated. These results indicate that upon LVX exposure, *P. aeruginosa* PAS71 enhanced many key genes involved in DNA replication and repair, and homologous recombination, which play an important role in repairing DNA damage caused by LVX. Therefore, these gene upregulations induced resistance to LVX in *P. aeruginosa* PAS71.

The three KEGG pathways shown in Supplementary Figures S1F–H are related to *P. aeiruginosa* virulence. Supplementary Figure S1F shows the *P. aeruginosa* biofilm formation pathway, in which many key genes were downregulated, indicating that LVX inhibited biofilm formation in PAS71. Supplementary Figure S1G shows the biosynthesis of siderophore group nonribosomal peptides pathway, in which several key genes were upregulated, indicating that these genes were enhanced by *P. aeruginosa* PAS71 upon LVX exposure. Figure 1H shows the bacterial secretion system pathway, in which many key genes involved in types II, III, and VI, and the Sec-SRP systems were upregulated, while few genes were downregulated, indicating that *P. aeruginosa* PAS71 enhanced these secretion systems upon LVX exposure. Therefore, LVX inhibited biofilm formation of *P. aeruginosa* PAS71; however, the biosynthesis of siderophore group nonribosomal peptides and types II, III, and VI, and the Sec-SRP bacterial secretion systems were enhanced upon LVX exposure.

Figure 4D shows 30 main KEGG pathways in which the up-downregulated genes of the PAS81 strain were enriched upon exposure to 0.125 μg/ml LVX. Almost all these pathways were enriched with upregulated genes, except one pathway of valine, leucine, and isoleucine degradation, which was enriched with both upregulated and downregulated genes. Of these 30 pathways, 1 pathway of homologous recombination was related to LVX resistance, 3 pathways (bacterial secretion system, *P. aeruginosa* biofilm formation, and biosynthesis of siderophore group nonribosomal peptides) were related to *P. aeruginosa* virulence, and the remaining 26 pathways were related to bacterial physiological metabolism. These results indicate that *P. aeruginosa* PAS81 enhanced DNA homologous recombination, some virulence factors, and physiological metabolism in response to LVX stress. Supplementary Figure S2 in Supplementary Material 2 shows four detailed KEGG pathways of the PAS81 RNA-seq, including homologous recombination (Supplementary Figure S2A), bacterial secretion system (Supplementary Figure S2B), biofilm formation (Supplementary Figure S2C), and biosynthesis of siderophore group nonribosomal peptides (Supplementary Figure S2D).

Supplementary Figure S2A shows the homologous recombination pathway, in which only the upregulated gene of *recA* was enriched, indicating that *P. aeruginosa* PAS81 enhanced *recA* expression in response to LVX stress. Supplementary Figure S2B shows the bacterial secretion system pathway, in which key genes involved in type II and VI systems were upregulated, indicating that *P. aeruginosa* PAS81 enhanced these secretion systems upon LVX exposure. Supplementary Figure S2C shows the *P. aeruginosa* biofilm formation pathway, in which upregulated key genes involved in type 6 bacterial secretion systems were enriched, and other key genes involved did not show differential expression. Supplementary Figure S2D shows the biosynthesis of siderophore group nonribosomal peptides pathway, in which one upregulated gene of *pchE* was enriched, indicating that *P. aeruginosa* PAS81 enhanced *pchE* expression upon LVX exposure.

#### qRT-PCR results

**Figure 5:**
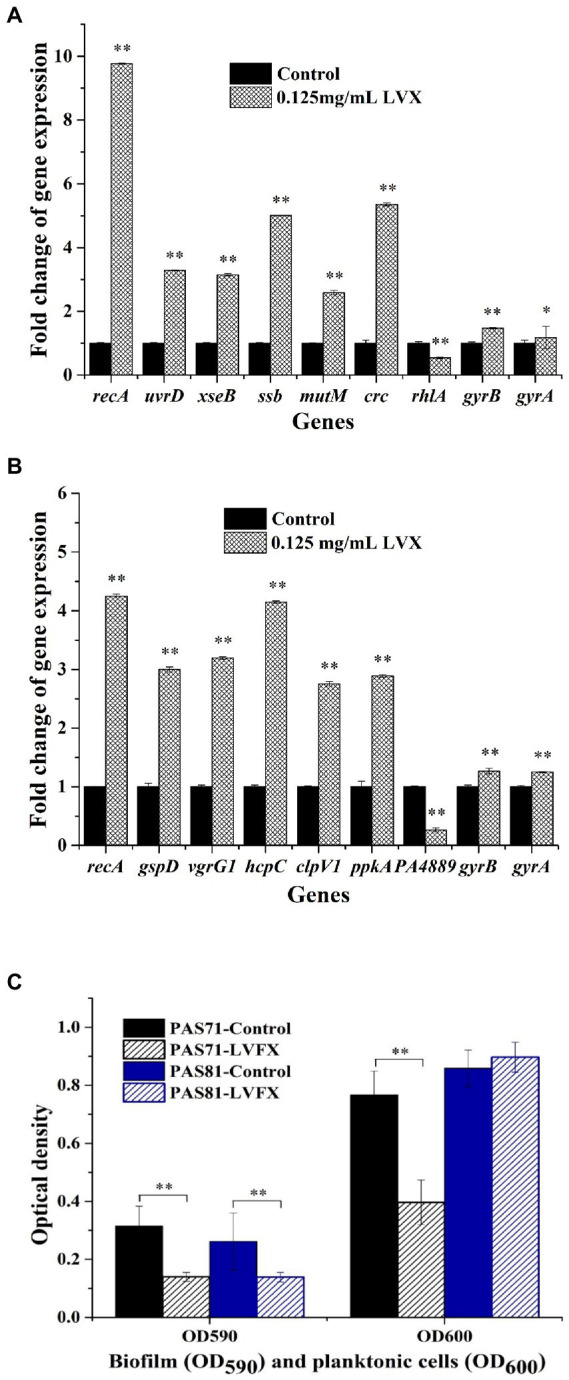
qRT-PCR and biofilm validation maps for *Pseudomonas aeruginosa* PAS71 and PAS81 strains. **(A)** qRT-PCR validation map for a selection of the differentially expressed key genes from PAS71 strain when exposed and unexposed to 0.125 μg/ml LVX for 5 h; **(B)** qRT-PCR validation map for the selection of differentially expressed key genes from PAS81 strain when exposed and unexposed to 0.125 μg/ml LVX for 5 h; **(C)** biofilm validation map for PAS71 and PAS81 strains exposed and unexposed to 0.125 μg/ml LVX for 24 h. *p < 0.05 based on an independent Student’s *t*-test; **p < 0.01.

The qRT-PCR validation maps of some key DEGs and two genes with unchanged transcriptional level (*gyrB* and *gyrA*) in *P. aeruginosa* PAS71 (Figure 5A) and PAS81 (Figure 5B) strains exposed to 0.125 μg/ml LVX are shown in Figure 5. The PAS71 qRT-PCR up-down expression of key genes shown in Figure 5A was consistent with that of transcriptomic analysis, where six genes were upregulated, including *recA* encoding recombinase RecA, *uvrD* encoding helicase II UvrD, *xseB* encoding exonucleases ExoVII, *ssb* encoding single-stranded DNA-binding protein SSB, *mutM* encoding bifunctional glycosylase Fpg, and *crc* encoding AP-endonuclease Xth; one gene *rhlA* encoding rhamnosyltransferase subunit A was downregulated; the expression of two genes, *gyrB* encoding DNA gyrase subunit B and *gyrA* encoding DNA gyrase subunit A, remained unchanged (fold change <2). The PAS81 qRT-PCR up-down expression of key genes shown in Figure 5B was consistent with that of transcriptomic analysis, where six genes were upregulated, including *recA* and five genes involved in bacterial secretion systems (*gspD*, *vgrG1*, *hcpC*, *clpV1*, and *ppkA*); one gene PA4889 encoding oxidoreductase was downregulated; the expression of two genes, *gyrB* and *gyrA*, remained unchanged. The transcriptional fold changes of all the determined DEGs in PAS71 and PAS81 were higher than two-fold. The qRT-PCR further confirmed the transcriptomic results and showed that the heteroresistance of *P. aeruginosa* PAS71 and PAS81 to LVX was not related to *gyrB* and *gyrA* transcriptional levels.

#### Biofilm results

The biofilm validation results for strains PAS71 and PAS81 are shown in Figure 5C. The OD_590_ and OD_600_ results represent the biofilm yield and planktonic cell density, respectively. Biofilm yields were decreased in both strains following exposure to 0.125 μg/ml LVX for 24 h (Figure 5C). The planktonic cell growth of PAS71 was inhibited following exposure to 0.125 μg/ml LVX, while that of the PAS81 was not. Therefore, LVX inhibited *P. aeruginosa* PAS71 and PAS81 biofilms and this inhibitory effects on biofilms were consistent with the transcriptomic results.

## Discussion

According to the previous heteroresistance definition (EI-Halfawy and Valvano, 2015; Andersson et al., 2019; Nicoloff et al., 2019), the experimental results of K–B, MIC, and PAP showed that with the exception of PAS72 and PAS82, which were susceptible to LVX with MICs of 0.25 and 0.5 μg/ml, respectively, all other experimental strains ATCC27853, PAO1, PAS71, and PAS81 were heteroresistant to LVX, with MICs of 0.5, 2, 0.25, and 1 μg/ml, respectively. However, as shown in the PAP curves, only the resistant subpopulation concentration at frequency ≥ 1 × 10^−7^ in the PAS81 strain reached clinical resistance level (≥ 4 μg/ml) at 4 μg/ml. In contrast, the resistant subpopulation concentrations at frequencies ≥1 × 10^−7^ in the strains ATCC27853, PAO1, and PAS71 were all lower than the clinical resistance level at 1, 2, and 1 μg/ml, respectively. This indicates that although *P. aeruginosa* is prone to developing heteroresistance to LVX, including the quality control strain ATCC27853, few of these heteroresistant strains’ resistant subpopulation can reach clinical resistance level. Nonetheless, we should not dismiss this phenomenon, as the resistance levels of these resistant subpopulations are likely to reach or exceed clinical resistance level under continuous antibiotic stress.

Antibiotic resistance usually causes bacterial growth defects or weakened virulence, called fitness costs, owing to production and maintenance of resistance machinery (Wang et al., 2019; Pi et al., 2020; Balbontín et al., 2021). Susceptible bacterial populations usually outcompete resistant ones in the absence of antibiotics because of the fitness cost (Pacheco et al., 2017). Nevertheless, the fitness cost resulting from mutations can be compensated by a secondary mutation, altered gene expression, or metabolic compensation (Millan et al., 2015; Pacheco et al., 2017). The growth fitness cost usually generates susceptible revertants from resistant strains in antibiotic-free conditions; however, it will not generate susceptible revertants owing to compensation. The fitness cost test indicates that all heteroresistant subpopulation isolates of *P. aeruginosa* PAS71 and PAS81 had a fitness cost in their growth rates, which can affect resistance stability.

The resistance stability of different bacterial strains to antibiotics varies. Polymyxin B shows both a stable mutational resistance and unstable adaptive resistance against *P. aeruginosa* (Tam et al., 2005). Another study reported that colistin-susceptible revertants were obtained from 5 of the 17 colistin-resistant mutants, including three *P. aeruginosa*, one *Acinetobacter baumannii*, and one *Escherichia coli* strain (Lee et al., 2015). Adaptive resistance is unstable, while mutational resistance is stable; nonetheless, the mutation may revert owing to the growth fitness cost. The resistance stability test showed that growth rates of all resistant subpopulation isolates of PAS71 and PAS81 were unstable, with their MICs reverting to 2 μg/ml from the initial 4 or 8 μg/ml after continuous passage without antibiotics for 50 generations. A comprehensive analysis of the experimental results from the fitness cost and resistant stability tests showed that the resistance of *P. aeruginosa* PAS71 and PAS81 heteroresistant subpopulations was unstable because of the growth fitness cost.

The unstable heteroresistance of *P. aeruginosa* PAS71 and PAS81 may not be associated with SNPs and InDels of their genomes. Bacterial resistance induced by SNP and InDel mutations is usually stable. Quinolone antibiotics, such as LVX, target DNA gyrase and DNA topoisomerase IV, two essential topoisomerase enzymes (Hooper and Jacoby, 2015). Gyrase is a heterotetramer comprising two GyrA and two GyrB subunits, catalyzing a DNA double-strand break, passing another DNA strand through the break, and resealing it (Hooper and Jacoby, 2015). Mutations in *gyrA* and *gyrB*, known as quinolone-resistance-determining regions, can cause bacterial resistance to LVX (Pi et al., 2020). The SNP and InDel analyses based on WGS showed that one nonsynonymous SNP and one InDel mutations occurred in *gyrB* (PA0004) and *gyrA* (PA3168) of the PAS81 strain, respectively. The same nonsynonymous SNP mutation of *gyrB* also occurred in the PAS82 strain but not in PAS71, ATCC27853, and PAO1 strains. The InDel mutation of *gyrA* was not present in PAS71, PAS82, ATCC27853, and PAO1 strains. The SNP and InDel mutations in *gyrB* and *gyrA* of the PAS81 strain may change the structure of DNA gyrase, while changes in one of the LVX targets may induce a change in the susceptibility of the PAS81 strain to LVX. However, the experimental results of K–B, MIC, and PAP showed that the dominant population of the PAS81 strain was susceptible to LVX. Moreover, PAS82 strain is susceptible to LVX and had the same nonsynonymous SNP in *gyrB* as PAS81 strain. Therefore, the heteroresistance of *P. aeruginosa* PAS71 and PAS81 to LVX was not associated with *gyrB* and *gyrA* mutations.

The unstable heteroresistance of *P. aeruginosa* PAS71 and PAS81 strains could be due to adaptive changes at the transcriptional level. After 0.125 μg/ml LVX treatment for 5 h, transcriptomic analysis revealed a total of 5,577 and 5,581 genes in *P. aeruginosa* PAS71 and PAS81 RNA-seq, respectively. Of these, 2,349 (1,223 upregulated and 1,126 downregulated) and 257 (241 upregulated and 16 downregulated) genes were significantly differentially expressed in the PAS71 and PAS81 strains, respectively. The experimental results indicate that the antibacterial effect of 0.125 μg/ml LVX on the PAS71 strain was much greater than that on the PAS81 strain, as this concentration was 1/2 MIC of PAS71 strain and 1/8 MIC of PAS81 strain.

LVX blocks the resealing of DNA double-strand break and serves as a barrier against the replication fork to move forward and induce double-strand DNA breaks (Hooper and Jacoby, 2015). KEGG enrichment analysis results of the DEGs indicated that LVX downregulates the key genes involved in *P. aeruginosa* biofilm formation for PAS71 and PAS81 strains, thereby decreasing the biofilm yield. This further indicates that *P. aeruginosa* heteroresistance to LVX is not related to the biofilm. KEGG enrichment analysis also showed that for PAS71 strain, upregulated essential genes were involved in important pathways and worked together against LVX stress. This includes five pathways related to LVX resistance: mismatch repair, base excision repair, DNA replication, homologous recombination, and nucleotide excision repair; two pathways related to *P. aeruginosa* virulence: biosynthesis of siderophore group nonribosomal peptides and bacterial secretion systems; and some pathways related to physiological metabolism. Compared to the PAS71 strain, *P. aeruginosa* PAS81 upregulated essential genes involved in LVX resistance related pathway of homologous recombination, virulence related pathways of bacterial secretion systems and biosynthesis of siderophore group nonribosomal peptides, and some pathways related to physiological metabolism. These results indicate that the response of *P. aeruginosa* PAS71 and PAS81 to LVX stress is similar and consistent. However, the upregulated resistance response gene number of the PAS81 strain (*recA* encoding recombinase RecA) was lower than that of the PAS71 strain owing to the LVX concentration used, which was 1/8 MIC of the PAS81 strain and 1/2 MIC of the PAS71 strain.

RecA, a multifunctional enzyme in bacteria, plays a key role in homologous recombination, recombination repair, and SOS response (Namsaraev et al., 1998). RecA recombinase, not only promotes homologous pairing and strand exchange but also triggers DNA repair and recombination, chromosome partitioning, and cell division (Supplementary Figure S1C). The helicase II UvrD, is used by bacteria to repair diverse types of DNA lesions by filling the DNA gap using the complementary strand as a template (Supplementary Figures S1A,E; Epshtein et al., 2014). The single-stranded DNA-binding protein SSB, binds preferentially to ssDNA with a high affinity independent of sequence and often provides protection for transiently formed ssDNA, as well as directs a large number of proteins to sites of DNA replication, recombination, and repair (Supplementary Figures S1A,C,D; Roy et al., 2009). The DNA polymerase III holoenzyme (Supplementary Figure S1D) is a complex enzyme containing 10 subunits. It is the most critical enzyme in DNA replication, extending primer chains and synthesizing DNA leading and subsequent chains (Kelman and O’Donnell, 1995). The DNA primase DnaG, as shown in Supplementary Figure S1D, often transfers the primer to DNA polymerase holoenzyme using the action of the SSB proteins (Greci et al., 2022). In addition, the DNA repair protein RecO, exonucleases ExoVII, single-stranded-DNA-specific exonuclease RecJ, bifunctional glycosylase Fpg, AP-endonuclease Xth, and monofunctional glycosylase MPG also act in DNA repair and homologous recombination (Supplementary Figures S1A–C). Therefore, *P. aeruginosa* PAS71 and PAS81 strains enhanced the key genes involved in DNA replication and repair, and homologous recombination to compensate for DNA replication inhibition caused by LVX, leading to resistance to LVX. Moreover, the transcriptomic and qRT-PCR results showed that the transcriptional levels of *gyrB* and *gyrA* were unchanged, indicating that the heteroresistance of PAS71 and PAS81 strains to LVX was not related to the expression levels of *gyrB* and *gyrA*. In addition, *P. aeruginosa* PAS71 and PAS81 strains enhanced some virulence factors and physiological metabolism in response to LVX stress, such as bacterial secretion systems and biosynthesis of siderophore group nonribosomal peptides.

Bacterial secretion systems are key virulence weapons for pathogenic bacteria that successfully evade human hosts. There are six protein secretion systems in gram-negative bacteria (type I–VI; Supplementary Figures S1H, S2B). Types I, II, and V secretion systems usually deliver virulence factors to the bacterial cell surface or host environment (Hachani et al., 2011). The type II secretion system of *P. aeruginosa* secretes virulence factors, such as exotoxin A and proteases LasA/LasB, to destroy human cells (Swietnicki et al., 2019). The types III and VI systems are used to disable and destroy the host′s immune system and destroy the host′s microbial flora, respectively (Swietnicki et al., 2019). *P. aeruginosa* type VI secretion systems help to fight other host bacteria for their ecological niche and play a role in the pathogenic process (Sana et al., 2016). The KEGG pathway for the bacterial secretion system in Supplementary Figure S1H shows that the transcriptional levels of many key genes involved in types II, III, and VI, and Sec-SRP secretion systems of the PAS71 strain were upregulated following exposure to LVX. Supplementary Figure S2B also shows that some key genes involved in types II and VI secretion systems of the PAS81 strain were upregulated following exposure to LVX. These results indicate that *P. aeruginosa* PAS71 and PAS81 strains enhanced their virulence weapons in response to LVX stress.

Siderophores are ferric iron chelators secreted by bacteria to survive in low iron environments. Siderophore is biosynthesized *via* two pathways—biosynthesis of non-ribosomal peptides pathway and the non-ribosomal peptides synthetase-independent pathway (Carroll and Moore, 2018). Additionally, siderophores are considered virulence factors that participate in the regulation of some virulence factors, such as exotoxin A and proteases PrpL and AprA (Albelda-Berenguer et al., 2019). In this study, both *P. aeruginosa* PAS71 and PAS81 strains enhanced the key genes involved in biosynthesis of siderophore group non-ribosomal peptides upon exposure to LVX (Supplementary Figures S1G, S2D). This indicates that *P. aeruginosa* PAS71 and PAS81 strains enhanced siderophore biosynthesis to chelate ferric iron for growth and enhance the related virulence factors in response to LVX stress.

**Figure 6:**
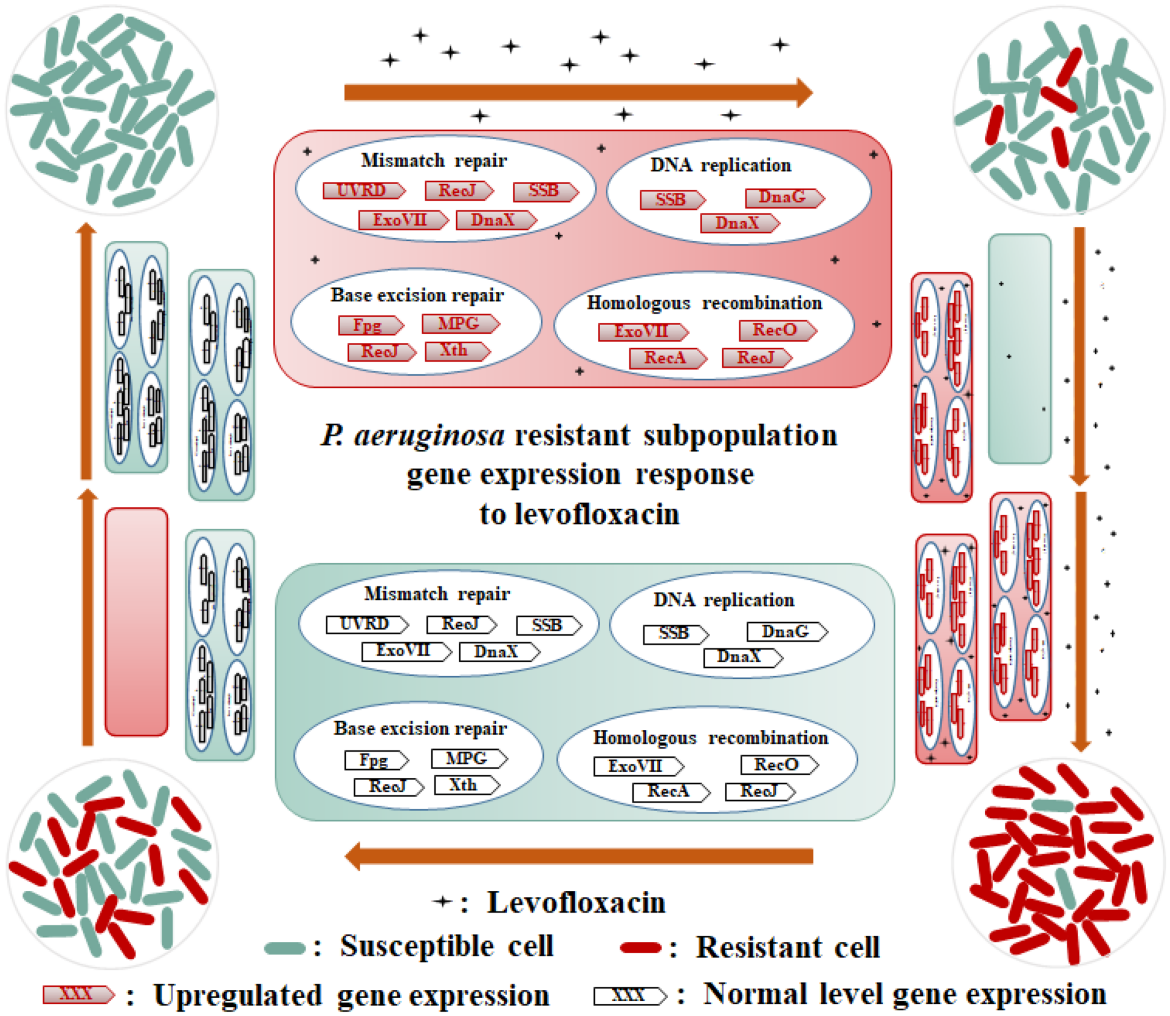
Schematic diagram of heteroresistance mechanism of *Pseudomonas aeruginosa* PAS71 and PAS81 strains to LVX. When exposed to LVX, the expression levels of some essential genes involved in DNA replication (SSB, DnaG, and DnaX), mismatch repair (UVRD, RecJ, SSB, ExoVII, and DnaX), base excision repair (Fpg, Xth, MPG, and RecJ), and homologous recombination (RecA, ExoVII, RecO, and RecJ) pathways of a few subpopulation cells of PAS71 and PAS81 strains susceptible to LVX are elevated; these subpopulations develop into LVX-resistant cells. After continuous exposure to LVX, the resistant subpopulation cells of the PAS71 and PAS81 strains selectively proliferate to become dominant populations. However, in the absence of antibiotic exposure, the expression levels of the essential genes involved in DNA replication and repair of a few resistant subpopulations revert to the parental strain levels.

Based on these analysis results, we generated a schematic diagram for the heteroresistant mechanism of *P. aeruginosa* strains toward LVX (Figure 6). When exposed to LVX, the expression levels of some essential genes involved in DNA replication and repair, as well as homologous recombination for some subpopulations of *P. aeruginosa* cells were elevated, including *recA* encoding recombinase RecA, *uvrD* encoding helicase II UvrD, *ssb* encoding single-stranded DNA-binding protein SSB, *dnaX* encoding DNA polymerase III subunits gamma/tau, r*ecO* encoding DNA repair protein RecO, *dnaG* encoding DNA primase DnaG, *xseA* and *xseB* encoding exonucleases ExoVII, *recJ* encoding single-stranded-DNA-specific exonuclease RecJ, *mutM* encoding bifunctional glycosylase Fpg, *crc* encoding AP-endonuclease Xth, and PA4010 encoding monofunctional glycosylase MPG. After continuous exposure to LVX, the resistant subpopulation cells of *P. aeruginosa* selectively proliferated to become dominant populations. However, in the absence of antibiotic exposure, the expression levels of essential genes involved in DNA replication and repair, and homologous recombination of a few resistant subpopulations reverted to the parental strain level.

## Conclusion

*Pseudomonas aeruginosa* PAS71, PAS81, ATCC27853, and PAO1 strains are heteroresistant to LVX with MIC values of 0.25, 1, 0.5, and 2 μg/ml, respectively; *P. aeruginosa* PAS72 and PAS82 strains are susceptible to LVX with MIC of 0.25 and 0.5 μg/ml, respectively. *P. aeruginosa* PAS71 and PAS81 strains were two typically different heteroresistant clinical isolates to LVX, with resistant subpopulation concentration of 1 and 4 μg/ml (the clinical resistance level ≥ 4 μg/ml), respectively, at frequency ≥ 1 × 10^−7^. The resistance of the PAS71 and PAS81 heteroresistant subpopulations were unstable and had a growth fitness cost. The unstable heteroresistance of PAS71 and PAS81 was caused by the elevated expression of essential genes involved in DNA replication and repair, and homologous recombination, rather than their genomic SNP and InDel mutations. Additionally, *P. aeruginosa* PAS71 and PAS81 enhanced some virulence factors and physiological metabolisms in response to LVX stress.

## Data availability statement

The datasets presented in this study can be found in online repositories. The names of the repository/repositories and accession number(s) can be found in the article/Supplementary material.

## Author contributions

W-RL: investigation, methodology, data analysis, writing – original draft, and funding acquisition. Z-QZ: methodology and visualization. KL: resources and data curation. B-BW, H-ZL, and Q-SS: visualization. X-BH: resources, data curation, and validation. X-BX: supervision and writing – review and editing. All authors contributed to the article and approved the submitted version.

## Funding

This work was supported by the Basic and Applied Basic Research Foundation of Guangdong Province (2021A1515011080), Guangdong Province Medical Science Research Foundation (A2018413), and GDAS’ Project of Science and Technology Development (2018GDASCX-0102).

## Conflict of interest

The authors declare that the research was conducted in the absence of any commercial or financial relationships that could be construed as a potential conflict of interest.

## Publisher’s note

All claims expressed in this article are solely those of the authors and do not necessarily represent those of their affiliated organizations, or those of the publisher, the editors and the reviewers. Any product that may be evaluated in this article, or claim that may be made by its manufacturer, is not guaranteed or endorsed by the publisher.

## Footnotes

^1^ https://www.ncbi.nlm.nih.gov/nuccore/NC_002516.2
