# Supplementary material for: Pseudomonas aeruginosa heteroresistance to levofloxacin caused by upregulated expression of essential genes for DNA replication and repair
Source: Front Microbiol. 2022 Dec 23;13:1105921. doi: 10.3389/fmicb.2022.1105921 (PMC9816134; doi:10.3389/fmicb.2022.1105921)
Supplement: Supplementary file 1 [file Data_Sheet_1.docx]

***Pseudomonas aeruginosa* heteroresistance to levofloxacin caused by upregulated expression of essential genes involved in** **DNA replication and repair**

Wen-Ru Li^1^, Zhi-Qing Zhang^1^, Kang Liao^2^, Bei-Bei Wang^1^, Hui-Zhong Liu^1^, Qing-Shan Shi^1^, Xu-Bin Huang^3*^, Xiao-Bao Xie^1^^*^

^1^Key Laboratory of Agricultural Microbiomics and Precision Application (MARA), Guangdong Provincial Key Laboratory of Microbial Culture Collection and Application, Key Laboratory of Agricultural Microbiome (MARA), State Key Laboratory of Applied Microbiology Southern China, Institute of Microbiology, Guangdong Academy of Sciences, Guangzhou, Guangdong, 510070, China

^2^Department of Clinical Laboratory, The First Affiliated Hospital of Sun Yat-sen University, Guangzhou, Guangdong, 510080, China

^3^Department of Pulmonary and Critical Care Medicine, The First Affiliated Hospital of Sun Yat-sen University, Guangzhou, Guangdong, 510080, China

Running title: *P. aeruginosa* heteroresistance to levofloxacin

^*^Corresponding author

Tel: +86-20-37656986, Fax: +86-20-37656986

E-mail: [xiexb@gdim.cn](mailto:xiexb@gdim.cn); [huangxb@mail.sysu.edu.cn](mailto:huangxb@mail.sysu.edu.cn)

Table S1 Statistics of the SNP annotation results of the five *P. aeruginosa* strains with *P. aeruginosa* PAO1 genome from NCBI database as the reference sequence

| **Strains** | **Type** | **Start_**  **syn** | **Stop_**  **syn** | **Start_**  **nonsyn** | **Stop_**  **nonsyn** | **Pre_**  **stop** | **Syn** | **Non_**  **syn** | **Total**  **_CDS** |
| --- | --- | --- | --- | --- | --- | --- | --- | --- | --- |
| *P. aeruginosa* PAS71 | number | 2 | 7 | 7 | 15 | 6 | 16604 | 5518 | 22145 |
| *P. aeruginosa* PAS81 | number | 3 | 14 | 15 | 29 | 12 | 29876 | 8540 | 38471 |
| *P. aeruginosa* PAS82 | number | 1 | 13 | 9 | 25 | 8 | 15471 | 6191 | 21705 |
| *P. aeruginosa* ATCC27853 | number | 3 | 9 | 7 | 7 | 7 | 16082 | 5231 | 21335 |
| *P. aeruginosa* PAO1 | number | 0 | 0 | 0 | 10 | 2 | 205 | 909 | 1125 |

Note: Start_syn, start codon synonymous mutation; Stop_syn, stop codon synonymous mutation; Start_nonsyn, start codon non-synonymous mutation; Stop_nonsyn, stop codon non-synonymous mutation; Pre_stop, a triplet codon mutated into a stop codon; Syn, synonymous in the coding region; Non-syn, non-synonymous in the coding region; Total_CDS, the total number of coding sequences with SNP mutation.

Table S2 Statistics of InDel type of the five *P. aeruginosa* strains with *P. aeruginosa* PAO1 genome from NCBI database as the reference sequence

| **Strains** | **Type** | **In_-**  **Start** | **In_**  **Middle** | **In_**  **Stop** | **Del_**  **Start** | **Del_**  **Middle** | **Del_**  **Stop** | **CDS_**  **InDel** |
| --- | --- | --- | --- | --- | --- | --- | --- | --- |
| *P. aeruginosa* PAS71 | Number | 2 | 52 | 1 | 1 | 61 | 0 | 74 |
| *P. aeruginosa* PAS81 | Number | 2 | 91 | 2 | 9 | 107 | 0 | 121 |
| *P. aeruginosa* PAS82 | Number | 3 | 67 | 1 | 1 | 78 | 2 | 76 |
| *P. aeruginosa* ATCC27853 | Number | 2 | 99 | 1 | 0 | 112 | 1 | 130 |
| *P. aeruginosa* PAO1 | Number | 1 | 18 | 1 | 0 | 11 | 0 | 20 |

Note: In_Start, insertion mutations located at the start codon; In_Middle, insertion mutations located at the middle of CDS; In_Stop, insertion mutations located at Stop Codon; Del_Start, deletion mutations located at the start codon; Del_Middle, deletion mutations located at the middle of CDS; Del_Stop, deletion mutations located at stop codon; CDS_InDel, the total number of coding sequences with InDel mutation.

Table S3 16 typical genes including one endogenous reference gene of *Pseudomonas aeruginosa* and their primer sequences used for real-time qPCR.

| **Locus tag** | **Gene symbol** | **Gene description** | **Primer sequence (5′-3′)** |
| --- | --- | --- | --- |
| PA5369.5 | PA5369.5 | 16S ribosomal RNA | GCGCAACCCTTGTCCTTAGTT (F)  TGTCACCGGCAGTCTCCTTAG (R) |
| PA3617 | *recA* | Recombinase A | GCCTGGTCATCTTCATCAA (F)  TTACCGTAGAGGATCTGGAA (R) |
| PA5443 | *uvrD* | DNA-dependent helicase II | CAGTTCAGCGACGACTTC (F)  GTTGGAGCGGTAGAGGAT (R) |
| PA4042 | *xseB* | Exodeoxyribonuclease VII small subunit | CTGCAAACCCTGGTGGAA (F)  GGAGGATCTGTACCTTCTGT (R) |
| PA4232 | *ssb* | Single-stranded DNA-binding protein | CTGGAAGGACAAGCAGAC (F)  ACGATCTCGGTGGTGTAG (R) |
| PA0357 | *mutM* | Formamidopyrimidine-DNA glycosylase | CCACTGGAACACGAGTTG (F)  ATGATGAACGGCTTGACC (R) |
| PA5332 | *crc* | Catabolite repression control protein | CAACTGGACGGCTACTTC (F)  TGGCGATACTCACCTTGT (R) |
| PA3479 | *rhlA* | Rhamnosyltransferase subunit A | CAGCAACCATCAGCACAT (F)  TCCAGGCAAGCCAAGTAG (R) |
| PA0685 | *gspD* | Type II secretion system protein | CTCCAACAGCATCGTGAT (F)  CGCAAGTAGACCACATGG (R) |
| PA0091 | *vgrG1* | Type VI secretion system protein VgrG | TGGAACAGGAAGGCATCTA (F)  GGAAGTCGTAGTCGTTGAG (R) |
| PA0263 | *hcpC* | Secreted protein Hcp | CCAGGAGCACTACTACAC (F)  ATCTTGCGGTAGGTGAAG (R) |
| PA0090 | *clpV1* | Secretion protein ClpV1 | GCGAGCAGAATGTCATCA (F)  GCCTTCTCCACTTCATCC (R) |
| PA0074 | *ppkA* | Serine/threonine protein kinase PpkA | GTGACCATCCACGACATC (F)  GAGTCCTTGCCGTTGTAG (R) |
| PA4889 | PA889 | Oxidoreductase | CTCCTGACCCCTTTGCTC (F)  ATAGTGGCGGTTCAACGA (R) |
| PA0004 | *gyrB* | DNA gyrase subunit B | GGCTGGTTGGAGAAGTTC (F)  ATTGCTGGCGAAGAAGTC (R) |
| PA3168 | *gyrA* | DNA gyrase subunit A | GCGAGCAGATTATCATCAC (F)  GCATAGAGGTTGTTGAGGA (R) |
