# Supplementary material for: Pseudomonas aeruginosa heteroresistance to levofloxacin caused by upregulated expression of essential genes for DNA replication and repair
Source: Front Microbiol. 2022 Dec 23;13:1105921. doi: 10.3389/fmicb.2022.1105921 (PMC9816134; doi:10.3389/fmicb.2022.1105921)
Supplement: Supplementary file 2 [file Data_Sheet_2.docx]

***Pseudomonas aeruginosa* heteroresistance to levofloxacin caused by upregulated expression of essential genes involved in** **DNA replication and repair**

Wen-Ru Li^1^, Zhi-Qing Zhang^1^, Kang Liao^2^, Bei-Bei Wang^1^, Hui-Zhong Liu^1^, Qing-Shan Shi^1^, Xu-Bin Huang^3*^, Xiao-Bao Xie^1^^*^

^1^Key Laboratory of Agricultural Microbiomics and Precision Application (MARA), Guangdong Provincial Key Laboratory of Microbial Culture Collection and Application, Key Laboratory of Agricultural Microbiome (MARA), State Key Laboratory of Applied Microbiology Southern China, Institute of Microbiology, Guangdong Academy of Sciences, Guangzhou, Guangdong, 510070, China

^2^Department of Clinical Laboratory, The First Affiliated Hospital of Sun Yat-sen University, Guangzhou, Guangdong, 510080, China

^3^Department of Pulmonary and Critical Care Medicine, The First Affiliated Hospital of Sun Yat-sen University, Guangzhou, Guangdong, 510080, China

Running title: *P. aeruginosa* heteroresistance to levofloxacin

^*^Corresponding author

Tel: +86-20-37656986, Fax: +86-20-37656986

E-mail: [xiexb@gdim.cn](mailto:xiexb@gdim.cn); [huangxb@mail.sysu.edu.cn](mailto:huangxb@mail.sysu.edu.cn)

**Figure S1**

**A**


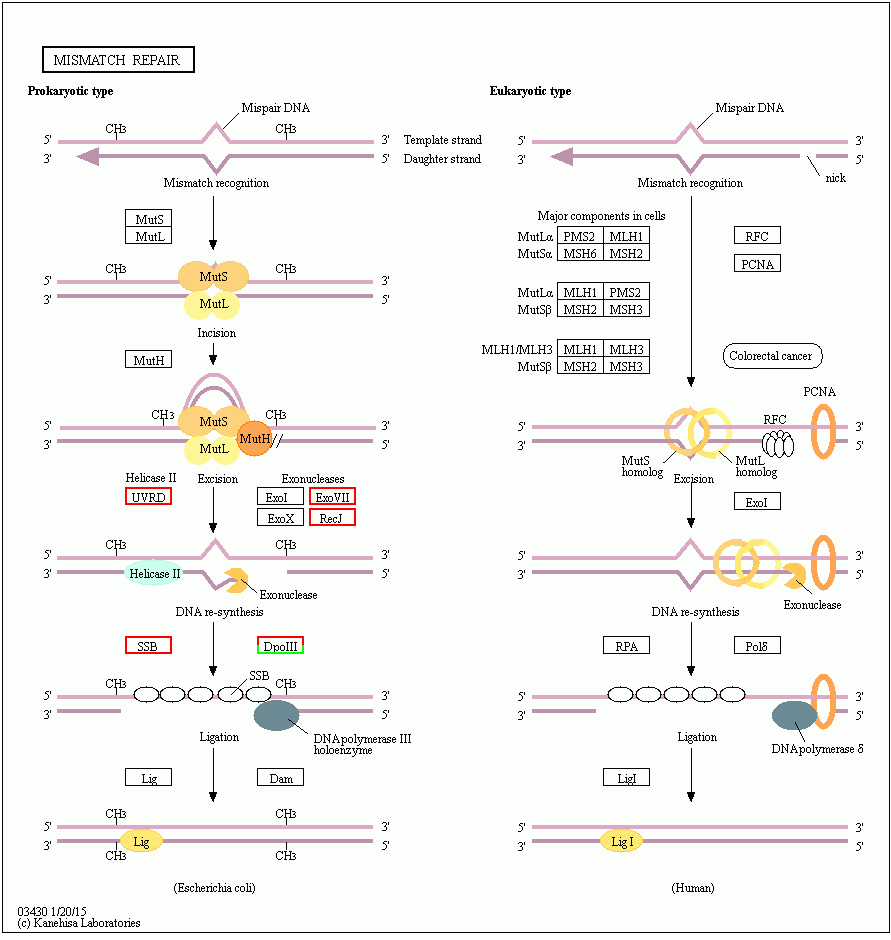


**B**


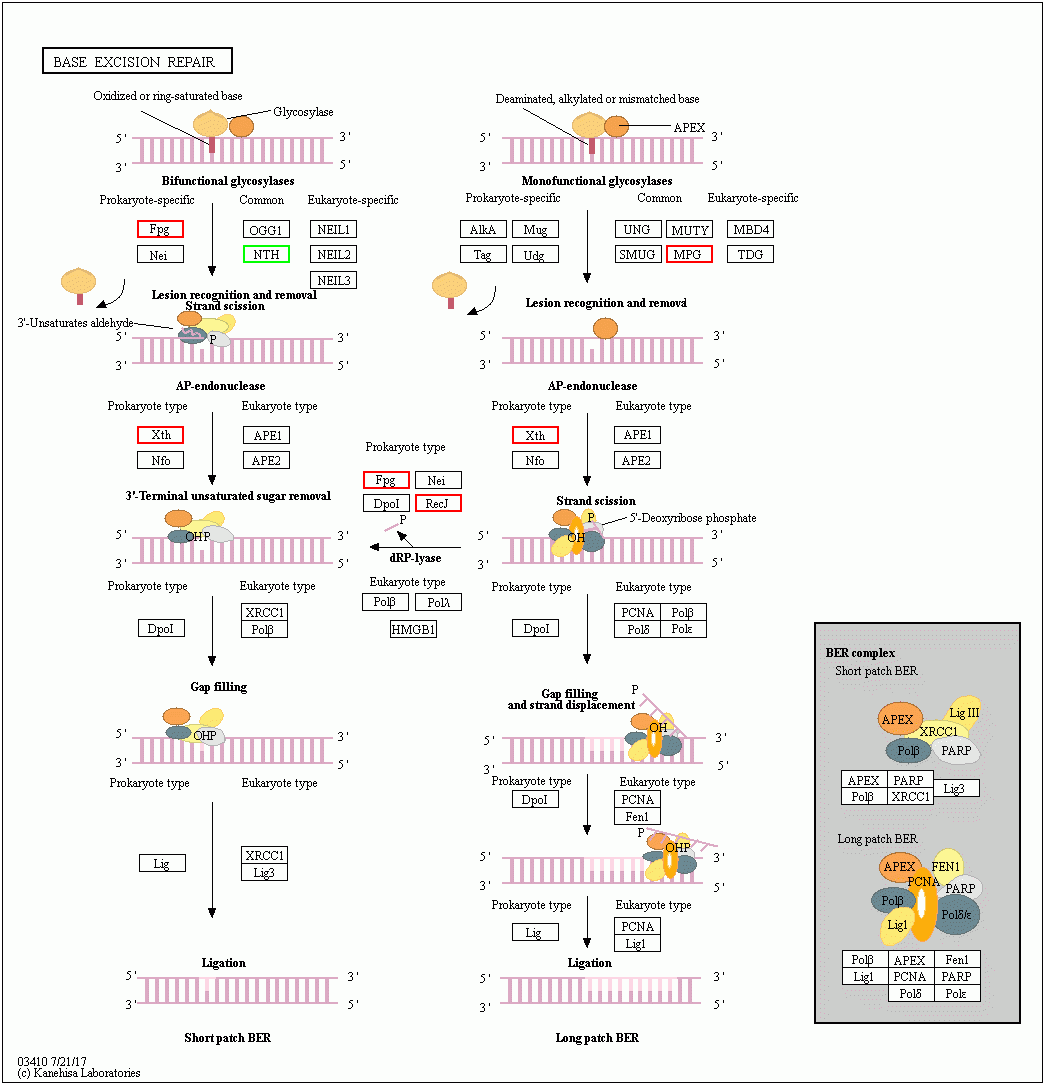


**C**


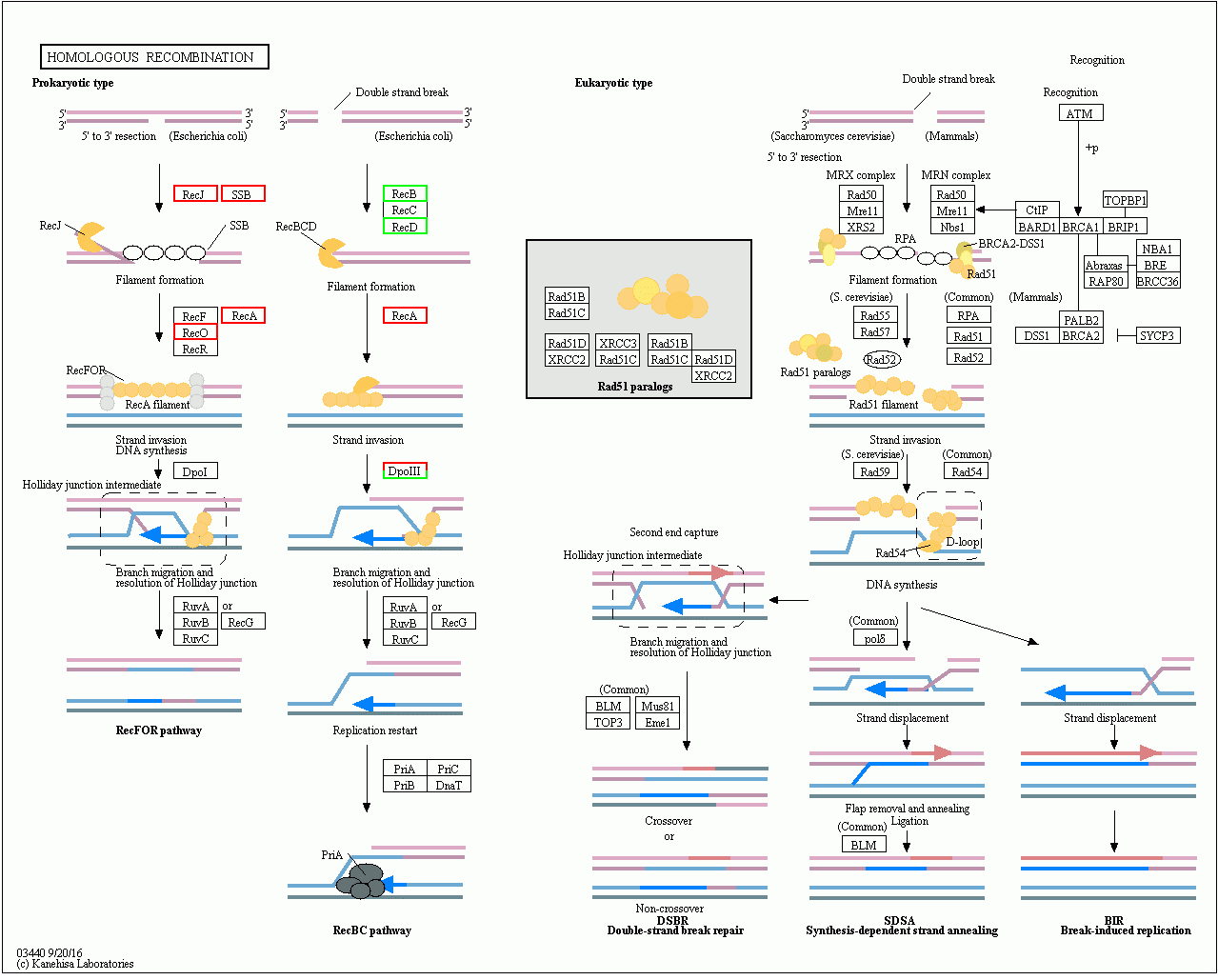


**D**


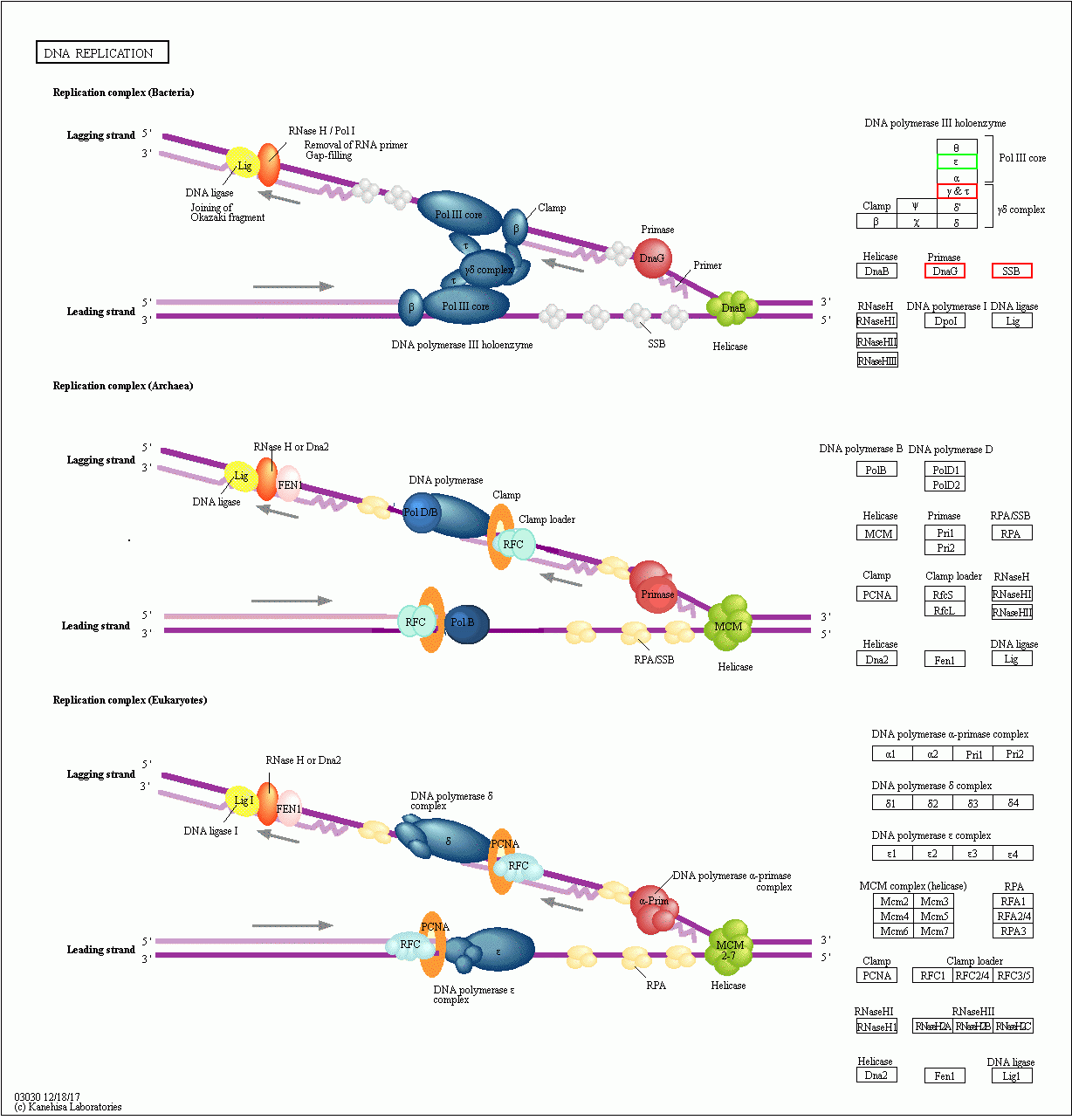


**E**


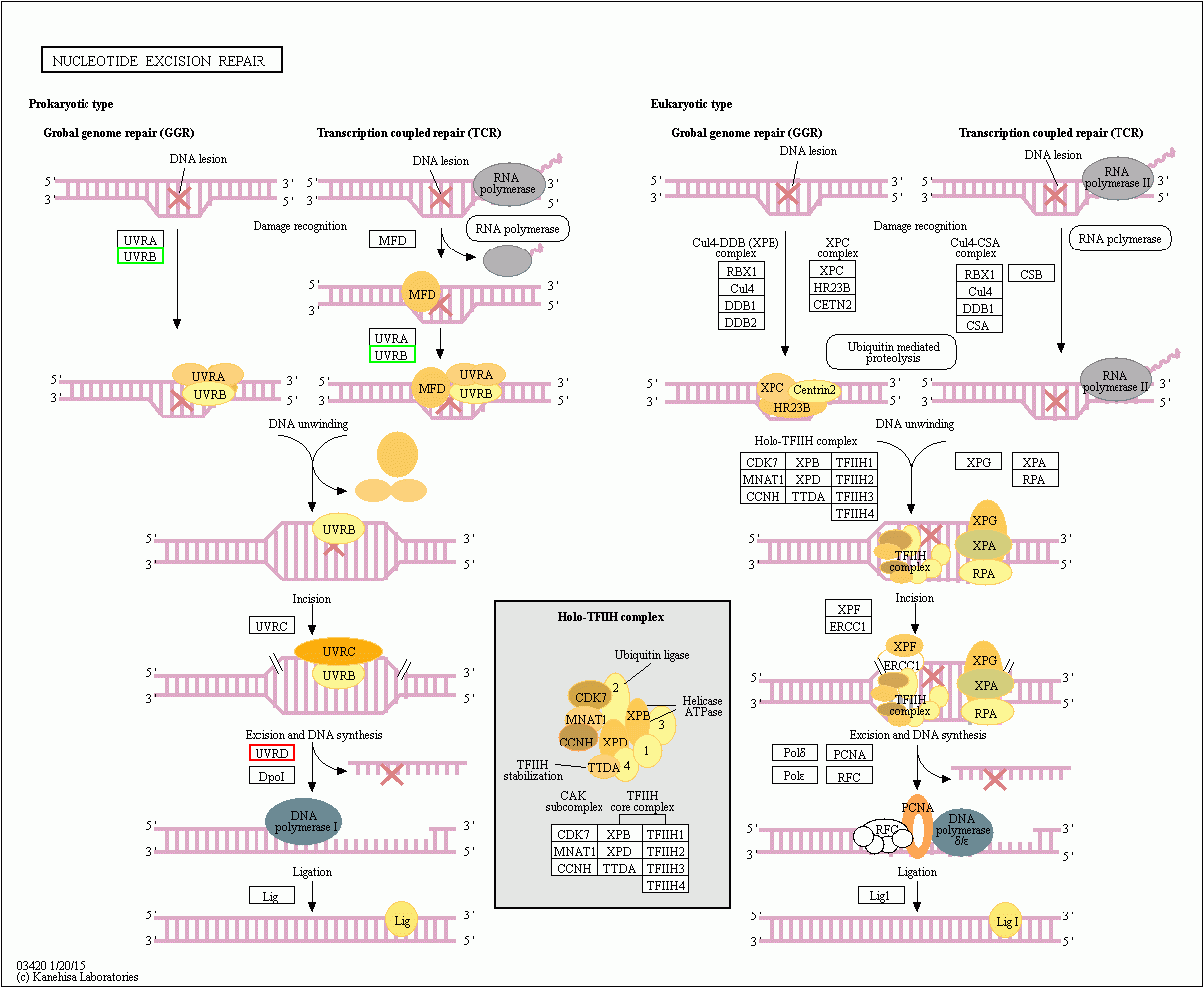


**F**


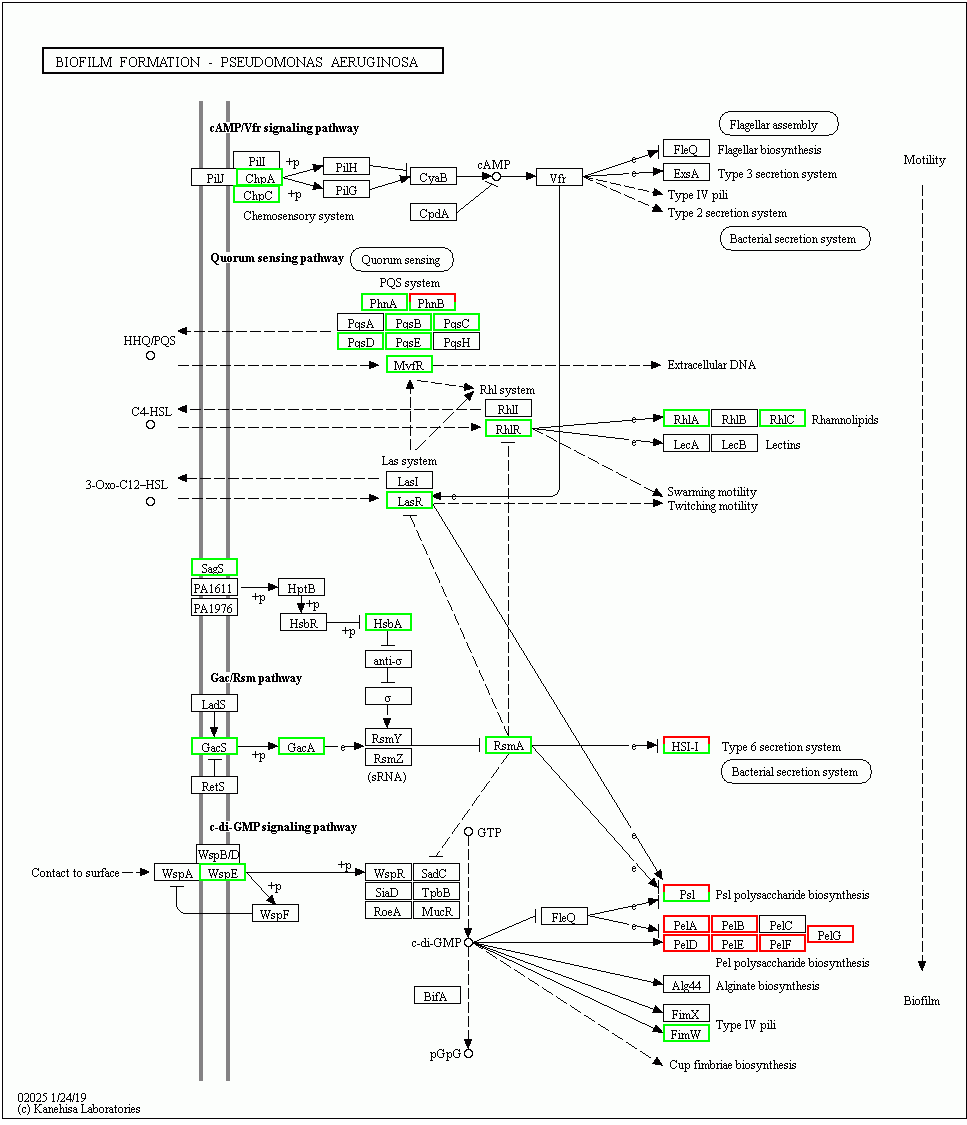


**G**


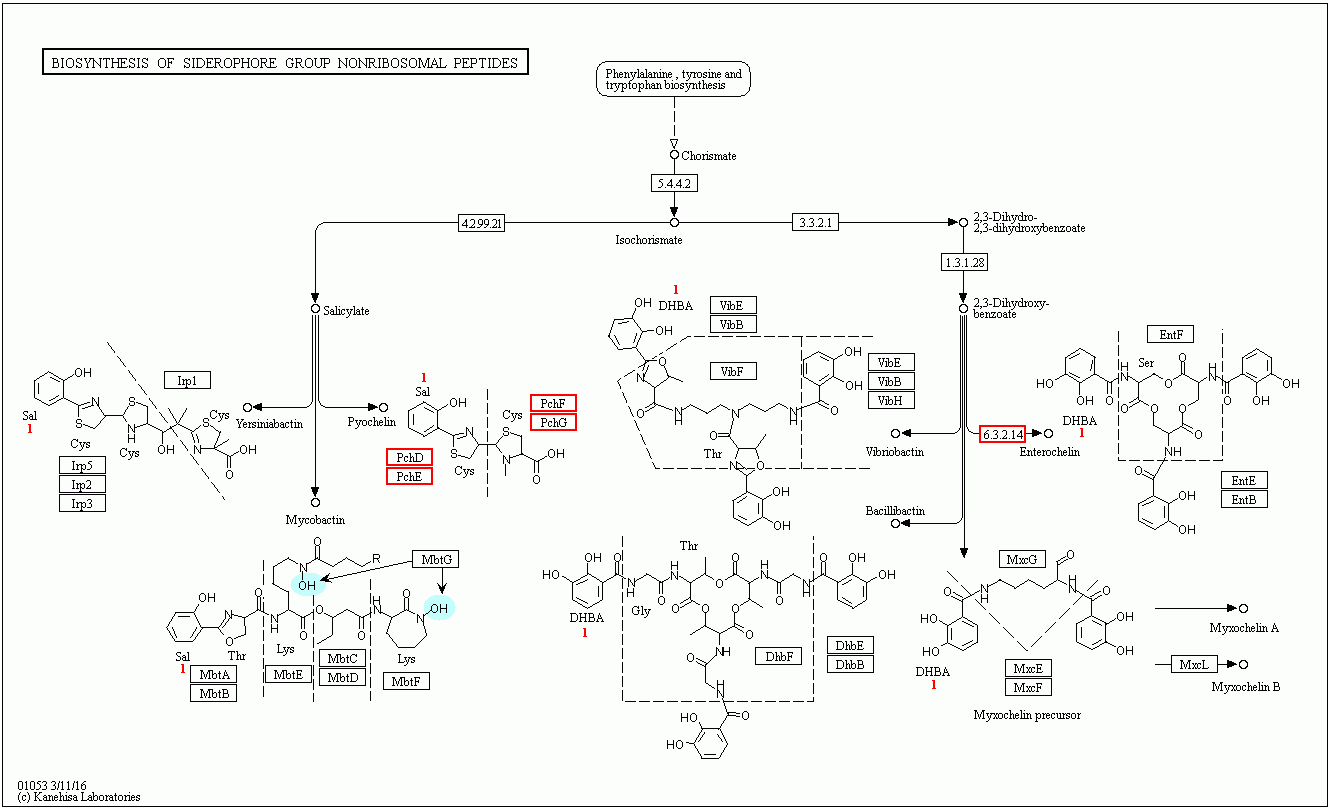


**H**


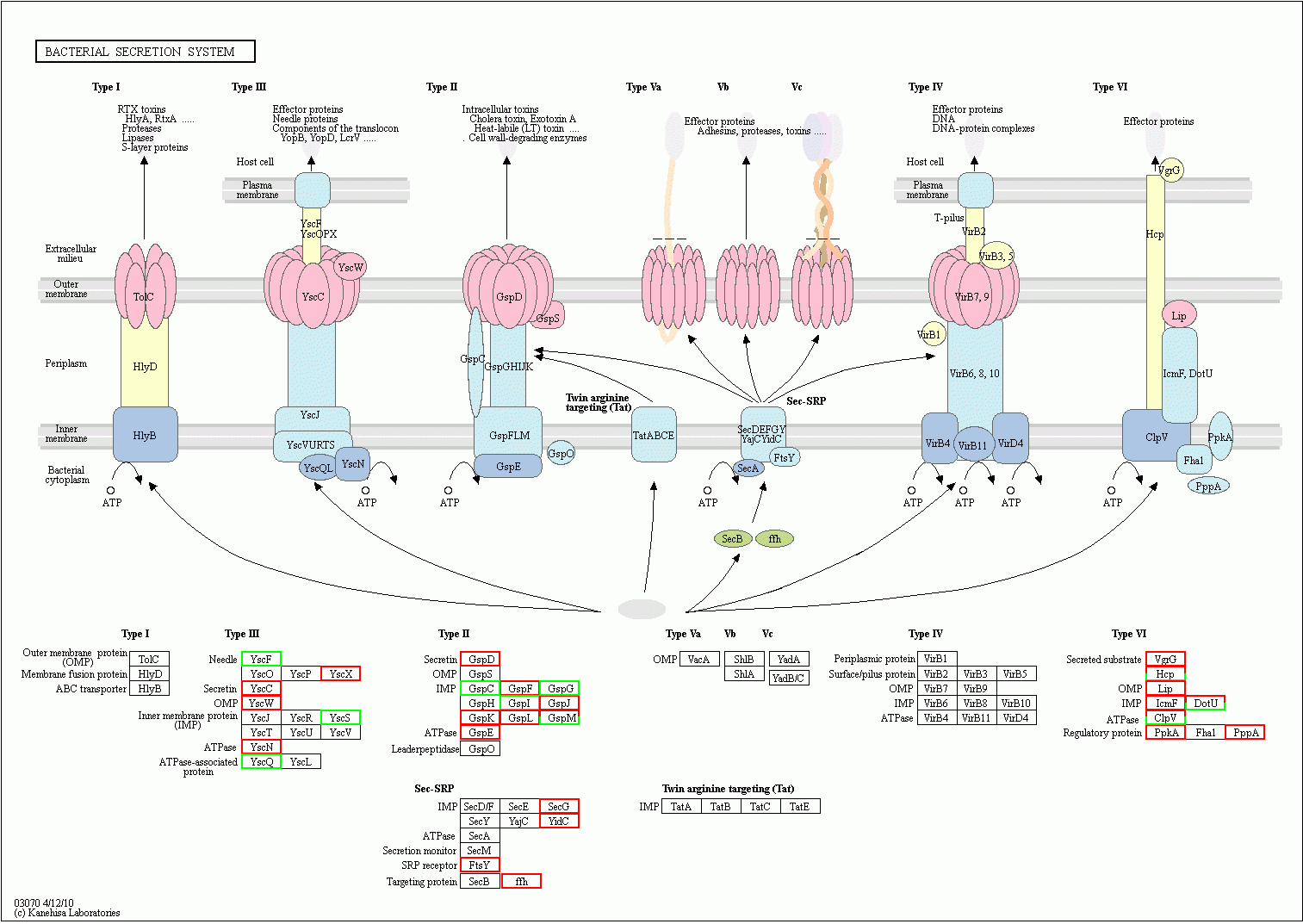


**Figure S1 The significantly enriched KEGG pathways based on transcriptomic data of the *Pseudomonas aeruginosa* PAS71 (Control group *vs* 0.125 µg/mL levofloxacin treated group). A, mismatch repair;** **B, base excision repair; C, DNA replication; D, homologous recombination;** **E, nucleotide excision repair;** **F, *P. aeruginosa* biofilm formation; G, biosynthesis of siderophore group nonribosomal peptides; H, bacterial secretion system.**

**Figure S2**

**A**


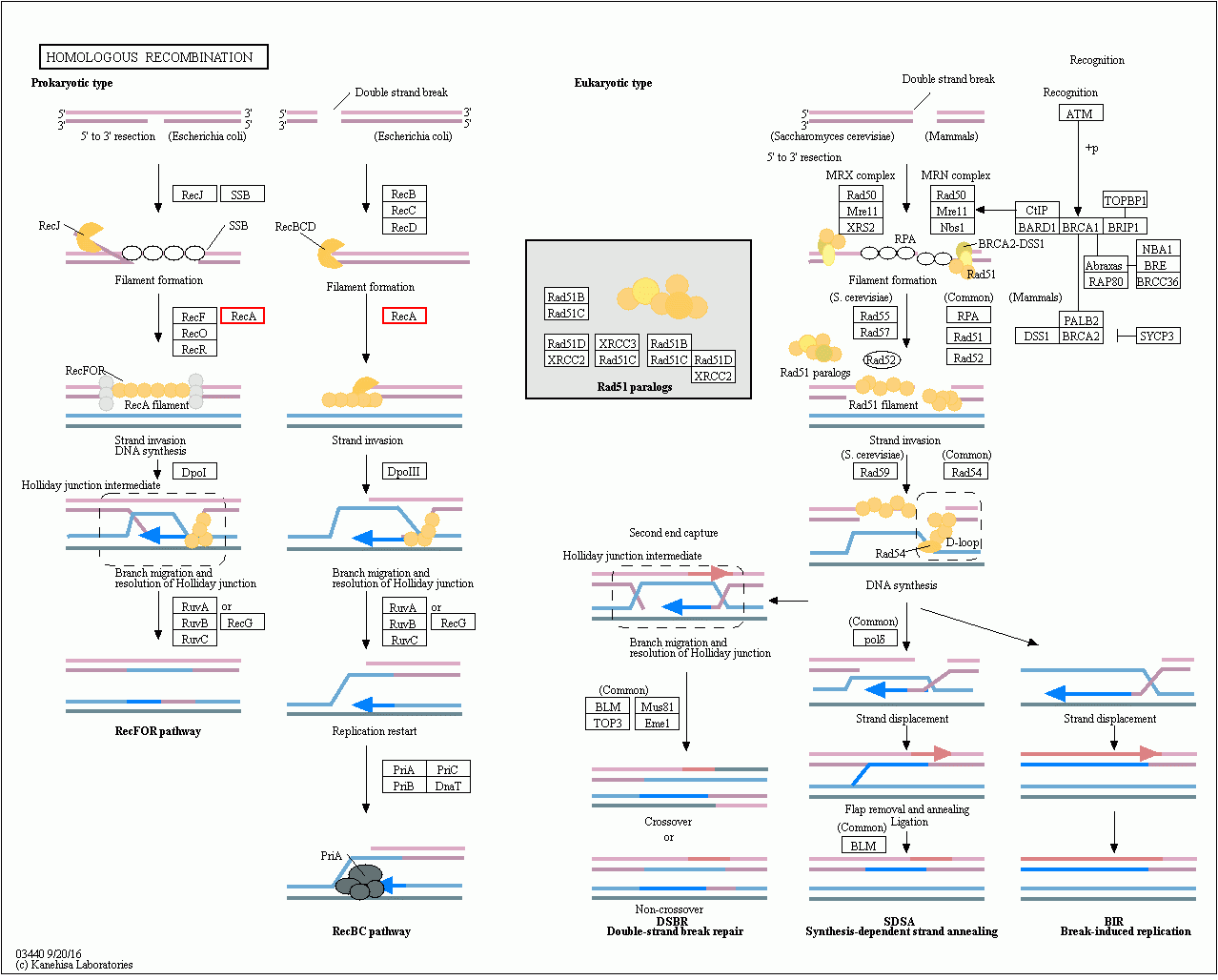


**B**


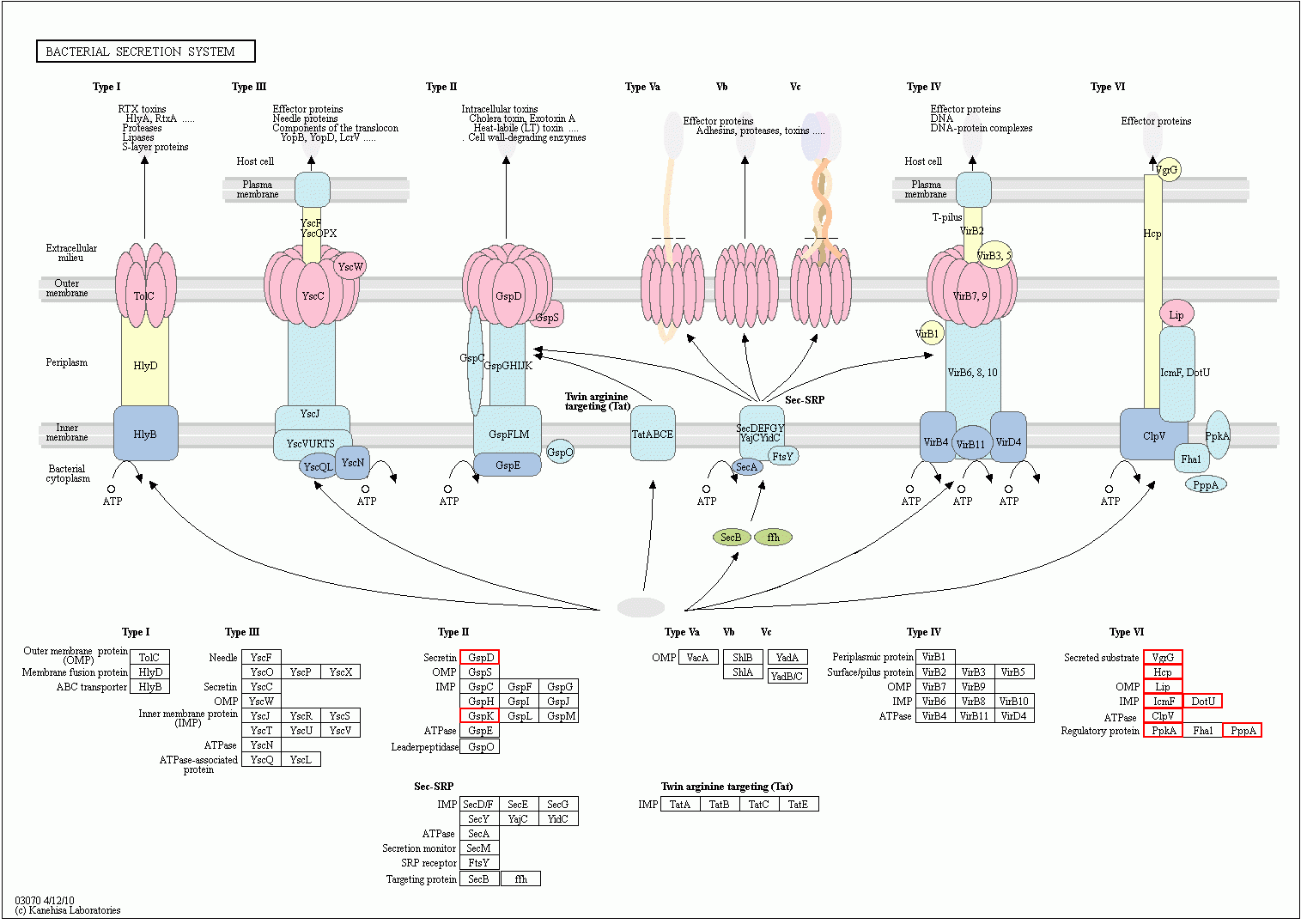


**C**


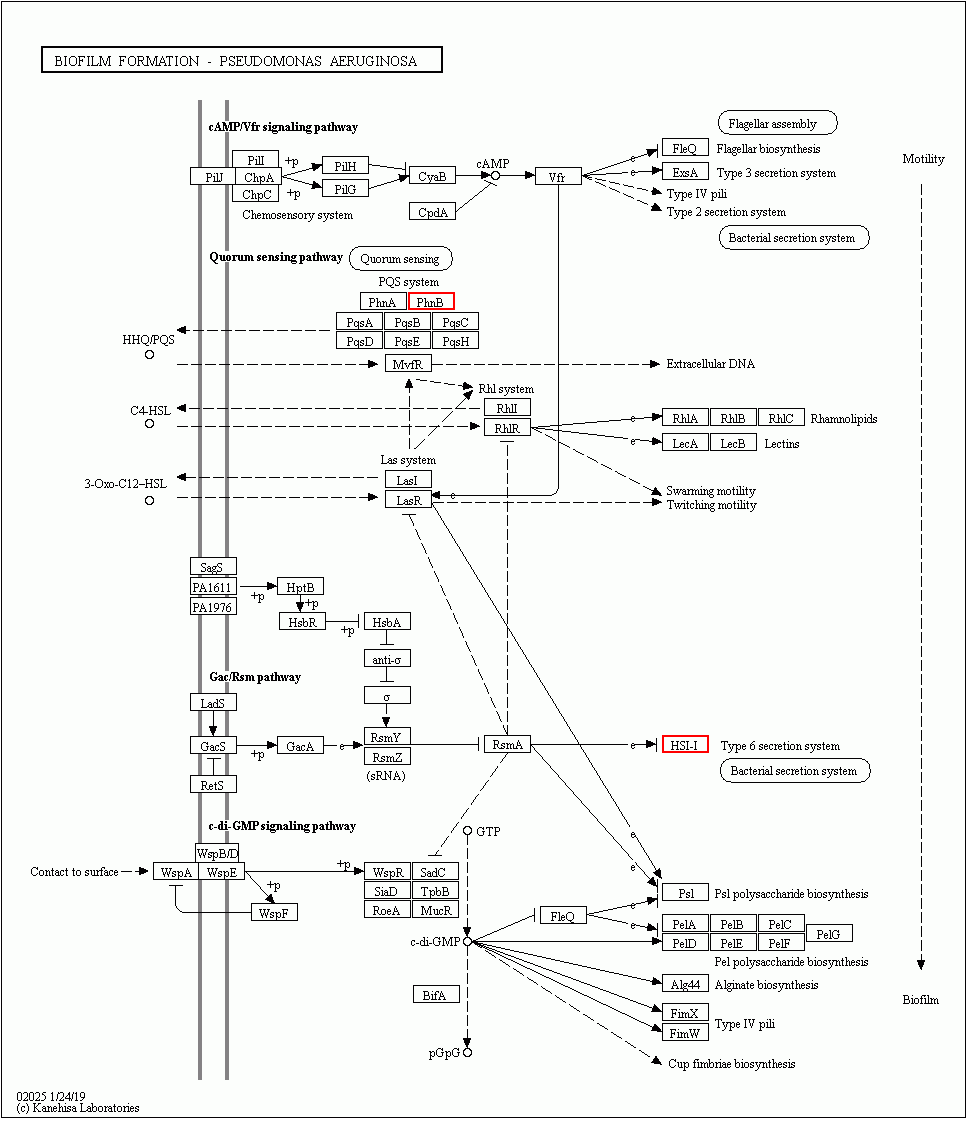


**D**


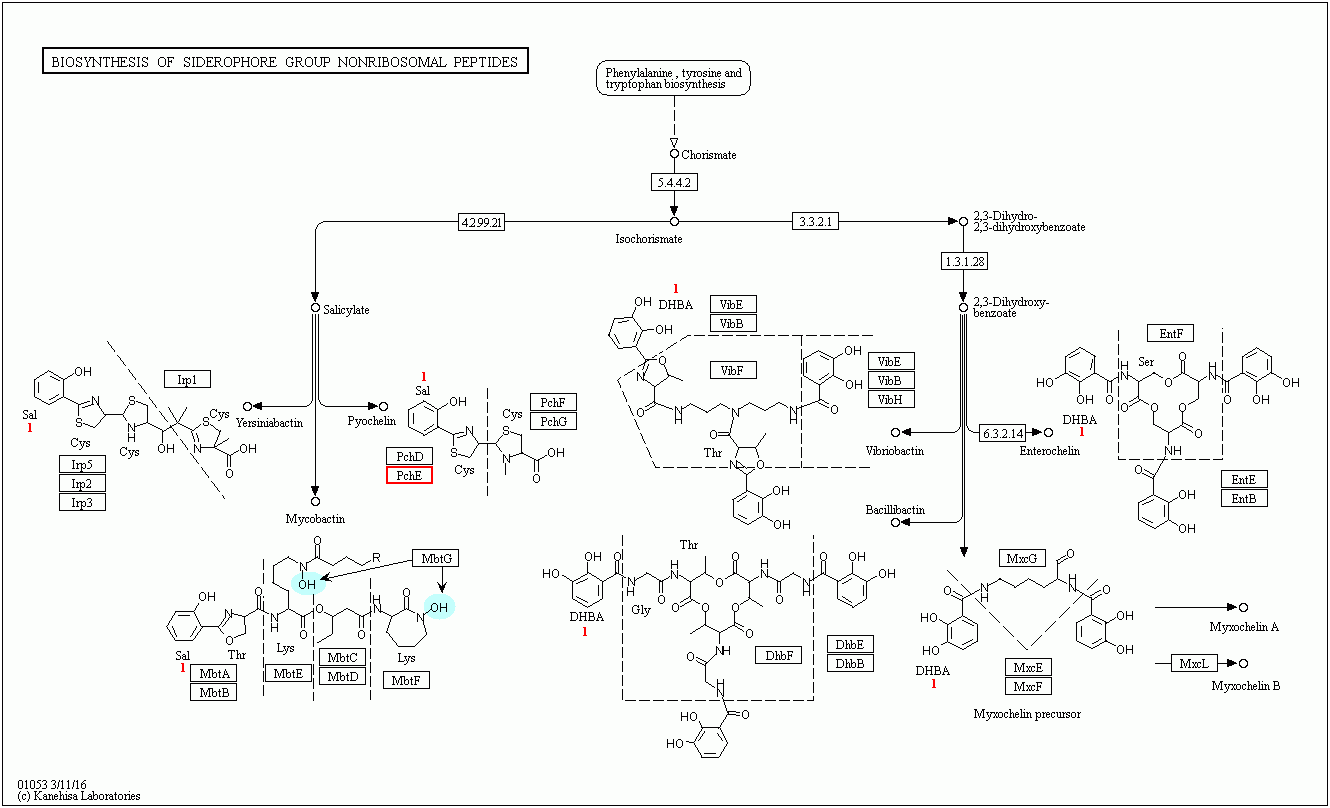


**Figure S2 The significantly enriched KEGG pathways based on transcriptomic data of the *Pseudomonas aeruginosa* PAS81 (Control group *vs* 0.125 µg/mL levofloxacin treated group). A, homologous recombination; B, bacterial secretion system; C, *P. aeruginosa* biofilm formation; D, biosynthesis of siderophore group nonribosomal peptides.**
